# Phosphate Transporter OsPT4, Ubiquitinated by E3 Ligase OsAIRP2, Plays a Crucial Role in Phosphorus and Nitrogen Translocation and Consumption in Germinating Seed

**DOI:** 10.1186/s12284-023-00666-9

**Published:** 2023-12-06

**Authors:** Yafei Sun, Fang Zhang, Jia Wei, Ke Song, Lijuan Sun, Yang Yang, Qin Qin, Shiyan Yang, Zhouwen Li, Guohua Xu, Shubin Sun, Yong Xue

**Affiliations:** 1 Institute of Eco-Environment and Plant Protection, Shanghai Academy of Agricultural Sciences, Shanghai, 201403 China; 2 State Key Laboratory of Crop Genetics and Germplasm Enhancement, Key Laboratory of Plant Nutrition and Fertilization in Low-Middle Reaches of the Yangtze River, Ministry of Agriculture, Nanjing Agricultural University, Nanjing, 210095 China; 3 Shanghai Key Laboratory of Protected Horticultural Technology, Shanghai Academy of Agricultural Sciences, Shanghai, 201403 China; 4 Key Laboratory of Crop Physiology, Ecology and Genetic Breeding, Ministry of Education, Jiangxi Key Laboratory of Crop Physiology, Ecology and Genetic Breeding, Jiangxi Agricultural University, Nanchang, 330045 China; 5 College of Fisheries and Life Science, Shanghai Ocean University, Shanghai, 201306 China; 6 Jilin Provincial Key Laboratory of Agricultural Biotechnology, Jilin Academy of Agricultural Sciences, Changchun, 130033 China

**Keywords:** Seed germination, *OsPT4*, Phosphate, Amino acid, Nitrogen

## Abstract

Phosphorus (P) and nitrogen (N) are essential macronutrients necessary for plant growth and development. OsPT4 is a high-affinity phosphate (Pi) transporter that has a positive impact on nutrient uptake and seed development. In this study, the expression patterns of different Pi transporter genes in germinating seeds were determined, and the relative expression of *OsPT4* was induced in Pi-deficient seeds and gradually increased with the passage of germination time. The analysis of P, N, Pi, and amino acid concentrations in germinating seeds of *OsPT4* mutants showed that the *OsPT4* mutation caused P and N retention and a continuous reduction in multiple amino acid concentrations in germinating seeds. Transcriptome analysis and qRT-PCR results also indicated that the *OsPT4* mutation inhibits the expression of genes related to P and N transportation and amino acid synthesis in germinating seeds. In addition, the paraffin section and TUNEL assay of *OsPT4* mutant germinating seeds suggests that *OsPT4* mutation causes programmed cell death (PCD) delayed in the aleurone layer and inhibition of leaf outgrowth. Moreover, we also found that OsPT4 was ubiquitinated by OsAIRP2, which is a C3HC4-type RING E3 Ub ligase. Our studies illustrate that *OsPT4* plays a crucial role in P and N collaborative translocation and consumption in germinating seeds. It also provides a theoretical basis for the molecules and physiological mechanisms of P and N cross-talk under suppressed Pi uptake conditions.

**Supplementary Information:**

The online version contains supplementary material available at 10.1186/s12284-023-00666-9.

## Introduction

Phosphorus (P) is an essential macronutrient necessary for plant growth and development, and a component of many important biological molecules, such as phospholipids, nucleic acids, P containing proteins and carbohydrates (Raghothama 1999; Wang et al. 2021a). Mined rock phosphate (Pi) is the predominant source of P fertilizers. Plants absorb, translocate and distribute Pi through Pi transporters in different tissues. These Pi transporters mainly belong to 2 protein families: the phosphate transporter (PHT) family and the SYG1/Pho81/XPR1 (SPX) domain-containing protein family (Wang et al. 2021b). Five PHT families, PHT1 (mainly located in the plasma membrane), PHT2 (located in chloroplasts), PHT3 (located in mitochondria), PHT4 (located in chloroplasts, the Golgi apparatus and nonphotosynthetic plastids) and PHT5 (located in vacuoles), have been identified in plants (Liu et al. 2016; Versaw and Garcia 2017). The original acquisition of Pi is primarily achieved by PHT1 (Shin et al. 2004). PHT1-family transporters use a Pi/H^+^ symport mechanism to take up Pi from the soil. This family consists of 9 and 13 members in Arabidopsis (*Arabidopsis thaliana*) and rice (*Oryza sativa*), respectively (Wang et al. 2021c). PHT1-family transporters have been demonstrated to mediate the acquisition of Pi from the soil environment and/or Pi translocation and distribution between cells or tissues.

The functions of PHT1 transporters have been generally discussed and reported (Jia et al. 2011; Ai et al. 2009; Zhang et al. 2015; Gu et al. 2016 et al. 2019; Dai et al. 2022). OsPT4 facilitates the development of rice embryos and the seed germination rate (Zhang et al. 2015). Seed germination is a key developmental process that transforms nondormant seeds into a highly active state and initiates the next stage of the plant life cycle (Rajjou et al. 2012). The process of seed germination includes three phases: imbibition, resumption of metabolic processes, and protrusion of the radicle through the seed envelope (Bewley 1997; Penfield et al. 2006; Holdsworth et al. 2008; Weitbrecht et al. 2011). Nitrogen (N) is the driving force of crop yields, and is also an abundant mineral nutrient for seed germination. The activation of amino acid biosynthesis and/or recycling pathways is essential for seed germination (Yobi et al. 2020). Reportedly, the contents of free amino acids are changed during the germination process (Fait et al. 2006; Dekkers et al. 2013; Galland et al. 2014; Angelovici et al. 2017). The levels of amino acids such as aspartate (Asp), threonine (Thr), and serine (Ser) increase significantly during the transition to germination in Arabidopsis seeds, along with a reduction in glycine (Gly), Gln, Ile, and the nonproteogenic r-aminobutyrate (Fait et al. 2006). Leucine (Leu), isoleucine (Ile), lysine (Lys), phenylalanine (Phe), tryptophan (Trp), tyrosine (Tyr) and threonine (Thr) can be degraded directly into acetyl-Co, which is the precursor for (GA) biosynthesis via hydroxymethylglutaryl CoA, to facilitate seed germination (Rios-Iribe et al. 2011). An increase in Pi (H_2_PO_4_^−^/HPO_4_^2−^) induces amino acids in rice seeds (Zhao et al. 2020). Therefore, the synergistic interaction and interaction between N and P are equally important for studying the mechanism of seed germination.

The coordinated absorption and transportation of N and P are crucial for plants to achieve nutrient balance and normal growth in unstable nutrient environments (Güsewell 2004; Khan et al. 2014; Luo et al. 2016). Many studies have confirmed that there interactions within crops during the absorption and utilization of N and P (Güsewell 2005). AtNRT1.1 and AtNIGT1 form a module for integrating nitrate and Pi signals. Through this module, changes in environmental N and P conditions can be sensed, thereby regulating root development through integrated commands (Medici et al. 2015). In addition, the expression of nitrate and Pi starvation activated AtNIGT1s is controlled by AtNLP7 and AtPHR1, which are central transcription factors for nitrate and Pi starvation signalling, thereby integrating AtNIGT1s into the main signalling pathways of N and P (Maeda et al. 2018). Several transporter genes can dramatically influence the homeostasis of N and P in both Arabidopsis and rice. For example, it has been reported that AtNRT1.5 is a nitrate transporter responsible for nitrate transport from roots to shoots, which is also involved in regulating the Pi starvation response of Arabidopsis (Cui et al. 2019). AtNRT1.1 also plays a role in the nitrate activated Pi deficiency response in an AtPHR1 dependent manner (Medici et al. 2019). In rice, nitrate promotes the synergistic absorption and utilization of N and P by regulating the binding of OsNRT1.1B (nitrate transporter) to the key negative regulatory factor SPX4 in the Pi signalling pathway, while activating the NLP3 and PHR2 dependent N and P signalling pathways in rice. However, the effect of Pi transporters on N translocation and assimilation was still not clear. Studying the effects of Pi transporters on N homeostasis can provide a more comprehensive understanding of the role of downstream transporters in N and P synergism.

Recent studies have shown that the abundance and activity of PHT1 are strictly regulated (Chen et al. 2015; Wang et al. 2018; Yang et al. 2020). The transcription factors Arabidopsis PHOSPHATE STARVATION RESPONSE 1 (PHR1) and rice PHR2 are involved in the Pi signalling pathway by binding to the PHR1-binding site (P1BS element) in the promoter of Pi transporters and other Pi starvation-responsive (PSR) genes (Rubio et al. 2001; Zhou et al. 2008; Puga et al. 2014; Wang et al. 2014; Zhong et al. 2018). In addition, PHT1 transporters are complete membrane proteins that are translated by ribosomes attached to the surface of the endoplasmic reticulum (ER) and that are folded and modified in the ER cavity before entering the final transport step of the plasma membrane through the membrane vesicle (Rodriguez-Furlan et al. 2019). PHOSPHATE TRANSPORTER TRAFFIC FACILITATOR 1 (PHF1) regulates the targeting of PHT1 proteins from the ER to plasma membranes in rice (González et al. 2005). Ubiquitin (Ub) is a small peptide that acts as a post-translational modifier to regulate virtually all aspects of cell biology in eukaryotes, including cell division, growth, communication, movement and death (Lee and Kim 2011). Ub is covalently linked to lysine (Lys) residues in other proteins by specific enzymatic cascades. These cascades begin with the transfer of a Ub moiety from an E1 Ub-activating enzyme (E1) to an E2 Ub-conjugating enzyme (E2). E3 Ub ligases (E3) represent the last step in the cascade, bringing together the E2 and the protein target that is then ubiquitinated (Smalle and Vierstra 2004). In Arabidopsis, NLA (NITROGEN LIMITATION ADAPTATION), which is a RING-type E3 Ub ligase, is the target of miR827, and miR399 mediates the degradation of Pht1 (Lin et al. 2013; Yang et al. 2020). OsNLA1 ubiquitinates two rice Pi transporters (OsPT2 and OsPT8), and eventually targets them for degradation in rice (Yue et al. 2017). Despite these studies, the regulatory mechanism of post-translational modification on Pht1 family members still needs further investigation. In our previous study, OsPT4 was shown to be a high-affinity Pi transporter that plays a pivotal role in Pi uptake and translocation, which is also significant for high Pi utilization in molecular breeding (Zhang et al. 2015). Because OsPT4 has very important application value in the regulation of P dynamic equilibrium and in the practical application of improving P utilization, its participation in the molecular regulation network research is also particularly important, however, at present, research on this aspect of *OsPT4* is still lacking.

In the current study, we report the significant function of *OsPT4* in programmed cell death (PCD) of the aleurone layer in germinating seeds and seedling leaf blade outgrowth. We found that the *OsPT4* mutation caused up-regulation of P and N accumulation and continuous reduction of multiple amino acid concentrations in germinating seeds. Transcriptome analysis and qRT-PCR detection illustrate that the *OsPT4* mutation inhibits the expression of genes related to P and N transportation and amino acid synthesis in germinating seeds. In addition, we also discovered that OsPT4 was ubiquitinated by the E3 Ub ligase OsAIRP2, which is homologous to AtAIRP2, a C3HC4-type RING E3 Ub ligase. Collectively, our data indicate that *OsPT4* plays a crucial role in P and N homeostasis and consumption in germinating rice seeds.

## Material and Methods

### Plant Materials and Growth Conditions

The *ospt4-1* (NE1260) and *ospt4-2* (SHIP_ZSF6267) mutants were obtained from the rice Tos17 insertion mutant database (https://tos.nias.affrc.go.jp/) and the Shanghai insertion population (http://ship.plantsignal.cn/), respectively. The rice (*Oryza sativa*) cultivar Nipponbare (the wild-type of *ospt4-1*) and Zhonghua11 (the wild-type of *ospt4-2*) were also used for experimental analysis. For hydroponic experiments, WT and mutant seeds were sterilized with 30% sodium hypochlorite (v/v) for 30 min, washed several times, and then incubated in sterile water at 30 ℃ in the dark until germination. Seeds were grown in nutrient solution (pH 5.5; refreshed every 3 days) with a day/night photoperiod of 16/8 h and a day/night temperature of 30 °C/24 °C, and the relative humidity was controlled at 60%.

### Determination of Total P, Pi, Amino Acid and GA Concentrations

For the determination of total P and Pi, a phosphomolybdate colorimetric assay was performed as described in Ames 1966.

For amino acid concentration determination, WT and *OsPT4* mutant seeds were grown for 3 and 7 days in nutrient solution (see above). The germinated seeds were collected and stored in liquid nitrogen for subsequent analysis of amino acid and hormone contents. The amino acid concentration was analysed using Agilent 1260 High-Performance Liquid Chromatography as described in (Palo Alto, CA, USA; Luo et al. 2018).

For hormone analysis, approximately 100 mg of frozen germinating seeds of WT and *OsPT4* mutants were extracted in 1 ml of ice-cold 50% aqueous ACN (vol/vol). After centrifugation (10 min, 12,000 rpm, 4 °C), the supernatant was transferred to clean plastic microtubes. All samples were purified using C18 reversed-phase, polymer-based, solid phase extraction (RP-SPE) cartridges, that had been washed with 1 ml of MeOH and 1 ml of deionized water, then equilibrated with 50% aqueous ACN (vol/vol). After loading a sample, the cartridge was then rinsed with 1 ml of 30% ACN (vol/vol) and this fraction was collected. The sample extracts were analyzed using an UPLC-Orbitrap-MS system (UPLC, Vanquish; MS, QE). The analytical conditions were as follows, UPLC: column, Waters ACQUITY UPLC HSS T3(1.8 μm, 2.1 mm*50 mm); column temperature, 40 °C; flow rate, 0.3 mL/min; injection volume, 2 μL; solvent system, water (0.1% Acetic acid): acetonitrile (0.1% Acetic acid); gradient program, 85:15 V/V at 0 min, 85:15 V/V at 0.5 min, 10:90 V/V at 1.5 min, 10:90 V/V at 3 min, 90:10 V/V at 3.1 min, 90:10 V/V at 5.0 min. HRMS data were recorded on a Q Exactive hybrid Q–Orbitrap mass spectrometer equipped with a heated ESI source (Thermo Fisher Scientific) utilizing the SIM MS acquisition methods. The ESI source parameters were set as follows: spray voltage, 3.0 kV; sheath gas pressure, 40 arb; aux gas pressure, 10 arb; sweep gas pressure, 0 arb; capillary temperature, 320 °C; and aux gas heater temperature, 350 °C.

### Transcriptome Analysis

The seeds of WT and mutants was harvested at 3-d after germination. Total RNA was extracted using Trizol reagent kit (Invitrogen, Carlsbad, CA,USA) according to the manufacturer’s protocol. After total RNA was extracted, eukaryotic mRNA was enriched by Oligo (dT) beads. The enriched mRNA was fragmented into short fragments using fragmentation buffer and reversly transcribed into cDNA by using NEB Next Ultra RNA Library PrepKit for Illumina (NEB#7530, New England Biolabs, Ipswich, MA,USA).The purified double-stranded cDNA fragments were end repaired, A base added, and ligated to Illumina sequencing adapters.The ligation reaction was purified with the AMPure XP Beads(1.0X).And polymerase chain reaction (PCR) amplified.There sulting cDNA library was sequenced using Illumina Novaseq6000 by Gene Denovo Biotechnology Co. (Guangzhou,China). Reads obtained from the sequencing machines includes raw reads containing adapters or low quality bases which will affect the following assembly and analysis. Short reads alignment tool Bowtie2 (version 2.2.8) was used for mapping reads to ribosome RNA (rRNA) database. The rRNA mapped reads then will be removed. The remaining clean reads were further used in assembly and gene abundance calculation. An index of the reference genome was built, and paired-end clean reads were mapped to the reference genome using HISAT2. 2.4 and other parameters set as a default. The mapped reads of each sample were assembled by using StringTie v1.3.1 in a reference-based approach. For each transcription region, a FPKM (fragment per kilobase of transcript per million mapped reads) value was calculated to quantify its expression abundance and variations, using RSEM software. Given FPKM(A) to be the expression of gene A, C to be number of fragments mapped to gene A, N to be total number of fragments that mapped to reference genes, and L to be number of bases on gene A. The FPKM method is able to eliminate the influence of different gene lengths and sequencing data amount on the calculation of gene expression. Therefore, the calculated gene expression can be directly used for comparing the difference of gene expression among samples.

Correlation analysis was performed by R. Correlation of two parallel experiments provides the evaluation of the reliability of experimental results as well as operational stability. The correlation coefficient between two replicas was calculated to evaluate repeatability between samples. The closer the correlation coefficient gets to 1, the better the repeatability between two parallel experiments. Principal component analysis (PCA) was performed with R package gmodels (http://www.r-project.org/) in this experience. PCA is a statistical procedure that converts hundreds of thousands of correlated variables (gene expression) into a set of values of linearly uncorrelated variables called principal components. PCA is largely used to reveal the structure/relationship of the samples/datas. RNAs differential expression analysis was performed by DESeq2[7] software between two different groups (and by edgeR between two samples). The genes/transcripts with the parameter of false discovery rate (FDR) below 0.05 and absolute fold change ≥ 2 were considered differentially expressed genes/transcripts.

Gene Ontology (GO) is an international standardized gene functional classification system which offers a dynamic-updated controlled vocabulary and a strictly defined concept to comprehensively describe properties of genes and their products in any organism. GO has three ontologies: molecular function, cellular component and biological process. The basic unit of GO is GO-term. Each GO-term belongs to a type of ontology. GO enrichment analysis provides all GO terms that significantly enriched in DEGs comparing to the genome background, and filter the DEGs that correspond to biological functions. Firstly all DEGs were mapped to GO terms in the Gene Ontology database (http://www.geneontology.org/), gene numbers were calculated for every term, significantly enriched GO terms in DEGs comparing to the genome background were defined by hypergeometric test. Genes usually interact with each other to play roles in certain biological functions. Pathway-based analysis helps to further understand genes biological functions. KEGG is the major public pathway-related database. Pathway enrichment analysis identified significantly enriched metabolic pathways or signal transduction pathways in DEGs comparing with the whole genome background.

### qRT-PCR

Total RNA (~ 1 µg) of WT (NP and ZH11) and mutant (*ospt4-1* and *ospt4-2*) 12 h, 24 h, 48 h and 72 h after germinated seeds were extracted TRIzol reagent (Invitrogen) for the synthesis of cDNA. The qRT-PCR analysis was performed by using 2 × TSINGKE Master qPCR Mix (SYBR Green II, TSINGKE) in the LightCycler 480 II (Roche). Relative expression levels of the genes were computed by the 2^−ΔΔCT^ method of relative quantification (Livak and Schmittgen 2001). The gene-specific primers used are listed in Additional file 2: Table S1.

### Paraffin Section and TUNEL Assay

For sectioning, the 3-d germinated seeds of WT and mutants were rinsed with plant tissue softening solution for 5–7 days. The immersed in deionized water and fixed in formalin/acetic acid/70% ethanol (1:1:18) for 72 h. Paraffin sectioning remaining steps refers to Ai et al. 2009. The Sections (15 mm thick) were transferred onto a slide and visualized using an Olympus (http://www.olympus-global.com/en/) BX51T stereomicroscope with a colour CCD camera.

The degradation of nuclear DNA was examined with terminal deoxynucleotidyl transferase (TdT)-mediated dUTP nick end labeling (TUNEL). Deparaffinize and rehydrate: incubate sections in 2 changes of xylene, 15–20 min each. Dehydrate in 2 changes of pure ethanol for 10 min each, followed by dehydrate in gradient ethanol of 95%, 90%, 80%, and 70% ethanol, respectively, 5 min each (extend deparaffinize time slightly in winter). Antigen retrieval: eliminate obvious liquid, mark the objective tissue with liquid blocker pen. Add proteinase K working solution to cover objectives and incubate at 37℃ for 25 min. then wash three times with PBS (pH 7.4) in a Rocker device, 5 min each. Permeabilization: eliminate excess liquid, add permeabilize working solution to cover objective tissue, then incubate at room temperature for 20 min. wash three times with PBS (pH 7.4) in a Rocker device, 5 min each. Equilibrium at room temperature: After the slices are slightly dried, buffer is added to the tissues in the circle, and the buffer is incubated at room temperature for 10 min. Tunel reaction: Take appropriate amount of TDT enzyme, dUTP and buffer in the tunel kit according to the number of slices and tissue size and mix at 1:5:50 ratio. Add this mixture to objective tissue placed in a flat wet box, incubate at 37 °C for 2 h. be sure to keep the wet box moist by adding water. DAPI counterstain in nucleus: wash three times with PBS (pH 7.4) in a Rocker device, 5 min each. Then incubate with DAPI solution at room temperature for 10 min, kept in dark place. Mount: wash three times with PBS (pH 7.4) in a Rocker device, 5 min each. Throw away liquid slightly, then coverslip with anti-fade mounting medium. Microscopic examination and collecting images through fluorescence microscope. DAPI emits blue light at an ultraviolet excitation wavelength of 330–380 nm and an emission wavelength of 420 nm; FITC has an excitation wavelength of 465–495 nm and an emission wavelength of 515–555 nm, and emits green.

### Yeast Two-Hybrid Screening and Yeast Two-Hybrid Assay

According to the user manual for the BD Matchmaker™ Library Construction and Screening Kit (Clontech, Mountain View, CA, USA), mRNA extracted from developing seeds (within 3 days after germination) was equally and evenly blended, then used for reverse transcription. The yeast two-hybrid (Y2H) screening was determined as described in Yan et al. 2023. A split-ubiquitin Y2H assay was performed using the DUAL membrane pairwise interaction kit (Dualsystems Biotech) according to the manufacturer’s instructions as described in Chang et al. 2019. The CDSs of *OsPT4* and *OsAIPR2* were cloned in frame into the vectors pBT3-N and pPR3-N via *Sfi* to generate Cub-Baits and NubG-Preys, respectively. The primers for the constructs are listed in Additional file 2: Table S1.

### BiFC Assays

To apply the bimolecular fluorescence complementation (BiFC) assays, synthetic coding DNA sequences (CDSs) of OsPT4 and OsAIPR2 were transferred from pENTR/D/TOPO to the pCAMBIA1300 vector, resulting in fusion with the yellow fluorescent protein C-terminus and N-terminus (cYFP and nYFP). These vectors were transformed into *Agrobacterium tumefaciens* strain EHA105, and the transformed *A. tumefaciens* were infiltrated into tobacco leaves and observed under a fluorescence microscope (Olympus FV1000) as described in Liu et al. 2010. The primers for the constructs are listed in Additional file 2: Table S1.

### E3 Ubiquitin Ligase Activity Assay

The full-length cDNA of *OsPT4* was cloned into the pcDNA3.1 vector, fused to the N-terminal myc-tag, and expressed in 293T cells. The myc-OsPT4 fusion protein was prepared according to the instruction manual (Amersham Biosciences, Piscataway, NJ, USA). For E3 ubiquitin ligase activity assay of the fusion proteins, the reaction containing recombinant wheat (Triticum aestivum) E1 (UBA1), human E2 (UBC8), E3 Os10g0445400 and ubiquitin were co-expressed in 293T cells. 293T cells were incubated with MG132 for 8 h before harvested, then added an appropriate amount of Co-IP-specific cell lysis buffer (containing protease inhibitors and 1 mM PMSF). Perform cell lysis on ice for 30 min. After cell lysis, centrifuged the cell lysate at maximum speed at 4 °C for 30 min, then collect the supernatant. After pre-clearing the lysis, protein G agarose beads were added at 10 μL per sample to the cell lysate. Incubate overnight with gentle shaking at 4 °C. Then removed the cell lysate, centrifuge at 1000 rpm for 5 min at 4 °C and pelleted the agarose beads at the bottom of the tube and removed the supernatant. Wash the beads for three times, add 2 × SDS loading buffer to the remaining agarose beads, boil in a water bath for 10 min. The anti-Ub antibody was used in Western-Blot assay.

### Statistical Analysis

All the data collected were analysed for significant differences using IBM SPSS Statistics version 23 software. Statistical analyses were performed by using Student’s test of one-way analysis of variance.

## Results

### The Expression Pattern of Phosphate Transporters in Germinating Seed

**Fig. 1:**
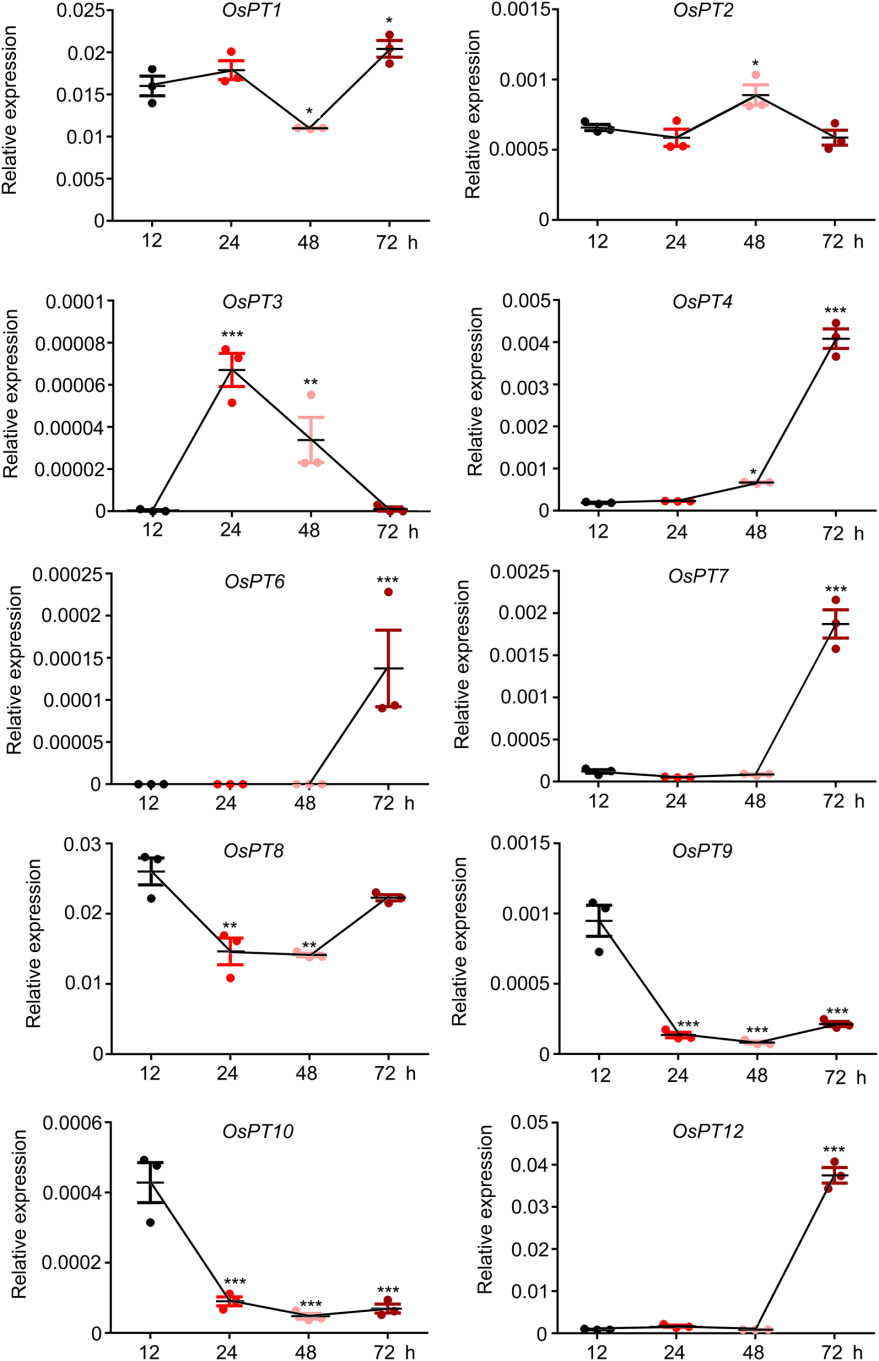
The relative expression of Pht1 family members in germinating rice seeds. The relative expression of *OsPT1*, *OsPT2*, *OsPT3*, *OsPT4*, *OsPT6*, *OsPT7*, *OsPT8*, *OsPT9*, *OsPT10* and *OsPT12* of NP seeds in 12 h, 24 h, 48 h and 72 h after germinated. Germinating seeds were harvested for qRT-PCR analysis. *OsACTIN* (LOC_Os10g36650) were used as internal controls. Values are means ± SE (*n* = 3). Asterisk indicate that the values differ significantly in comparisons with the expression level of *OsPT4* germinating for 12 h (*P < 0.05, **P < 0.01, ***P < 0.001, Student’ s test)

PHT1 family members are probably the only influx transporter for plant Pi uptake. Initially, we assessed the relative expression of Pht1 family members in germinating rice seeds to study the roles of Pi transporters during seed germination. In the course of our experiment, most of the seeds germinated and formed a sprouting protuberance from 48 to 72 h after germination. In the germinating seeds (germinated after 12 h, 24 h, 48 h, and 72 h), the relative expression of Pht1 family members represented different trends with the passage of germination time (Fig. 1). The relative expression of *OsPT2*, *OsPT3*, *OsPT6, OsPT9* and *OsPT10* was extremely low during germination. The relative expression level of *OsPT2* is relatively stable, reaching a peak at 48 h of germination. The relative expression of *OsPT3* was much higher in 24 h after germination seed than others. *OsPT6* was not expressed in seeds before 48 h of germination. After germinated for 48 h, the expression level of *OsPT6* gradually increasing in seeds. The expression pattern of *OsPT9* and *10* was quiet similar. Both of their relative expression pattern was sharp decreased after 12 h of germination (Fig. 1). In contrast, *OsPT1*, *OsPT4*, *OsPT7, OsPT8* and *OsPT12* appeared to be highly abundant. Among these genes, the variation trends of the relative expression of *OsPT1* and *OsPT8* during seed germination were relatively flat, falling first and then rising, and always maintaining a relatively high level of relative expression. This indicates the essential function of *OsPT1* and *OsPT8* in the process of seed germination. The relative expression of *OsPT4*, *OsPT7* and *OsPT12* increased rapidly from 48 to 72 h after germination. In contrast to *OsPT7* and *OsPT12*, the expression of *OsPT4* also showed a significant upward trend between 24 and 48 h after germination (Fig. 1). Seed germination process has been shown to begin with the imbibition of water (0–24 h), experiencing reboot and recovery of metabolic processes (24–48 h), and finally end with the protrusion of the coleoptile and radicle (48–72 h) (Bewley 1997; Penfield et al. 2006; Holdsworth et al. 2008; Weitbrecht et al. 2011). Combined with this process, it can be perceived that *OsPT4* continues to play functions in the process of sprouting protuberance (48–72 h of germination), from metabolic recovery to the protrusion of the coleoptile and radicle.

To further explore the expression pattern of Pi transporter genes responding to Pi deficiency in germinating seeds, seeds of NP in normal (NS) and low Pi (LPS) soil conditions were harvested for germination and qRT-PCR analysis (Additional file 1: Figure S1). We chose *OsPT1*, *OsPT4* and *OsPT8*, which are more abundant than other Pi transporter genes in seeds. The relative expression of *OsPT4* and *OsPT8* appeared to be significantly induced in LPS compared with NS, while *OsPT1* showed no change (Additional file 1: Figure S1). This result clearly shows that the relative expression level of *OsPT4* in LPS was up-regulated by 1.5-fold, while that of *OsPT8* was up-regulated by 0.5-fold. This indicates that *OsPT4* is the most strongly induced by low Pi in seeds among the three relatively abundant Pi transporter genes.

### Mutation of *OsPT4* Facilitates P and N Retention in Germinating Rice Seeds

**Fig. 2:**
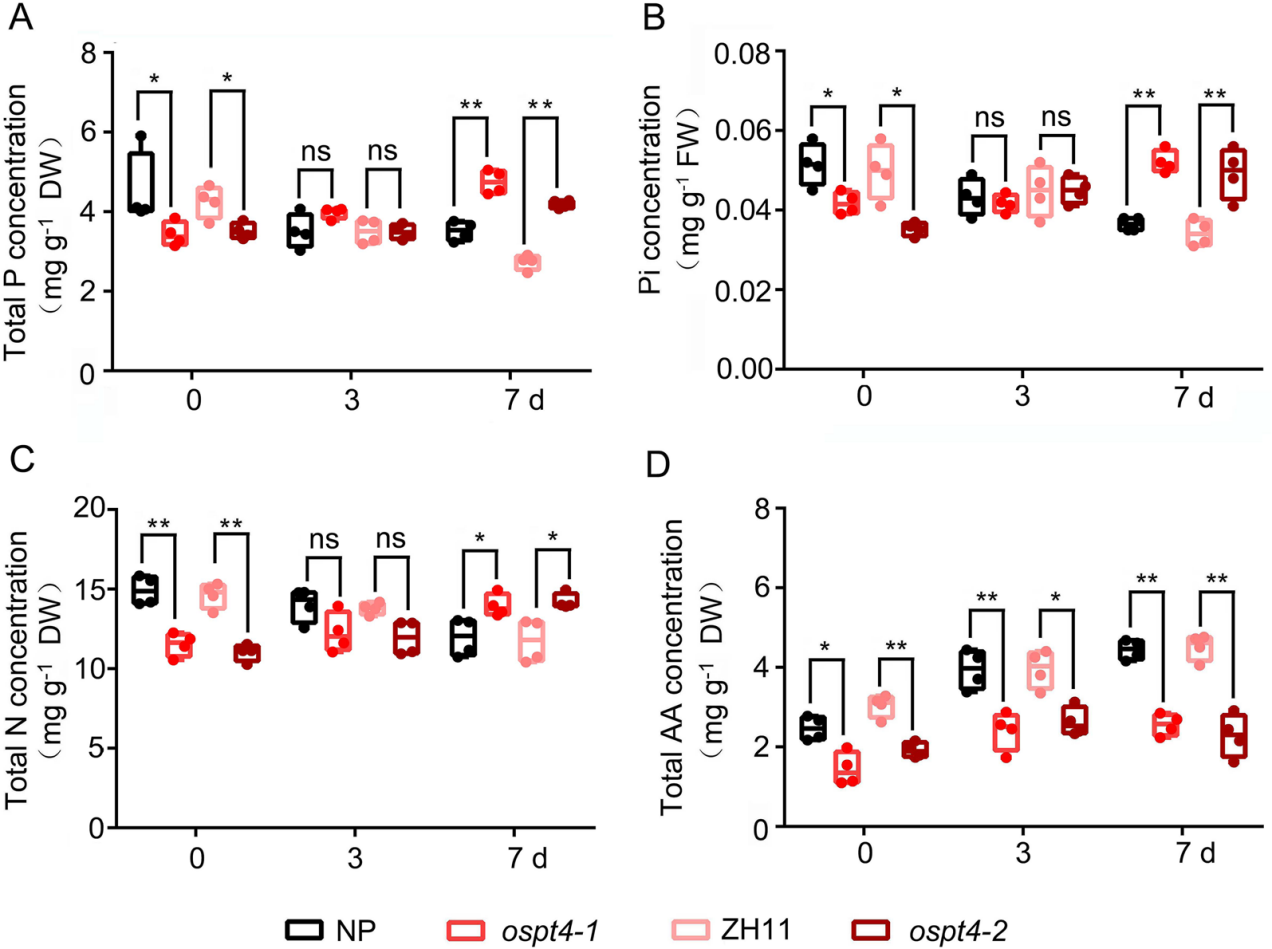
The mutation of *OsPT4* caused N and P retention in germinating seeds. **A**–**D** The concentration of total P (**A**), Pi (**B**), total N (**C**), and total amino acid (**D**) concentration in *OsPT4* mutants. The seeds of WT (NP and ZH11) and *OsPT4* mutants (*ospt4-1* and *ospt4-2*) germinated 0, 3, and 7 days were harvested for assaying the concentrations of total P, Pi, total N and total amino acid. *AA* amino acid. Values are means 4 replicates, ± SE (*n* = 4). Each replicates used 10 seeds. Different letters on the histograms indicate that the values differ significantly (*P < 0.05; **P < 0.01; Student’s t-test)

Two complete homozygous *OsPT4* knockout mutants (*ospt4-1* and *ospt4-2*) with a Tos17 and T-DNA insertion were validated by quantitative RT‒PCR and semiquantitative RT‒PCR and used for germinating seed morphology identification (Additional file 1: Figure S1). The primers for mutant identification are listed in Additional file 2: Table S1. To assess the effects of *OsPT4* mutation on nutrient accumulation and metabolism in germinating rice seeds, the total P, Pi, total N and total amino acid concentrations were determined in seeds of the WT and *OsPT4* mutants at 0, 3 and 7 d after germination (Fig. 2). As expected, the total P, Pi, N and amino acid concentrations in *OsPT4* mutants were significantly decreased at 0 d after germination (Fig. 2A–D). Interestingly, after germinating for 3 d, the total P, Pi and total N concentrations in germinating seeds were not changed in the mutants compared with the WT (Fig. 2A–C). The total P, Pi and total N concentrations were significantly increased in *OsPT4* mutant germinating seeds after germinating for 7 d (Fig. 2A–C). OsPT4 was a high-affinity Pi transporter that affects Pi uptake, translocation from roots to shoots (Zhang et al. 2015). We speculate that *OsPT4* may also act as a Pi transporter responsible for the redistribution of Pi from seeds to other vegetative organs during seed germination. This indicates that the *OsPT4* mutation may inhibit P and N translocation from seed to shoot and cause P and N retention in germinating seeds, leading to a gradual increase in P and N concentrations compared to those in the WT after germination. Nevertheless, throughout the entire germination process, the total amino acid concentrations were much lower in germinating seeds of the *OsPT4* mutants than in germinating seeds of the WT (Fig. 2D).

**Fig. 3:**
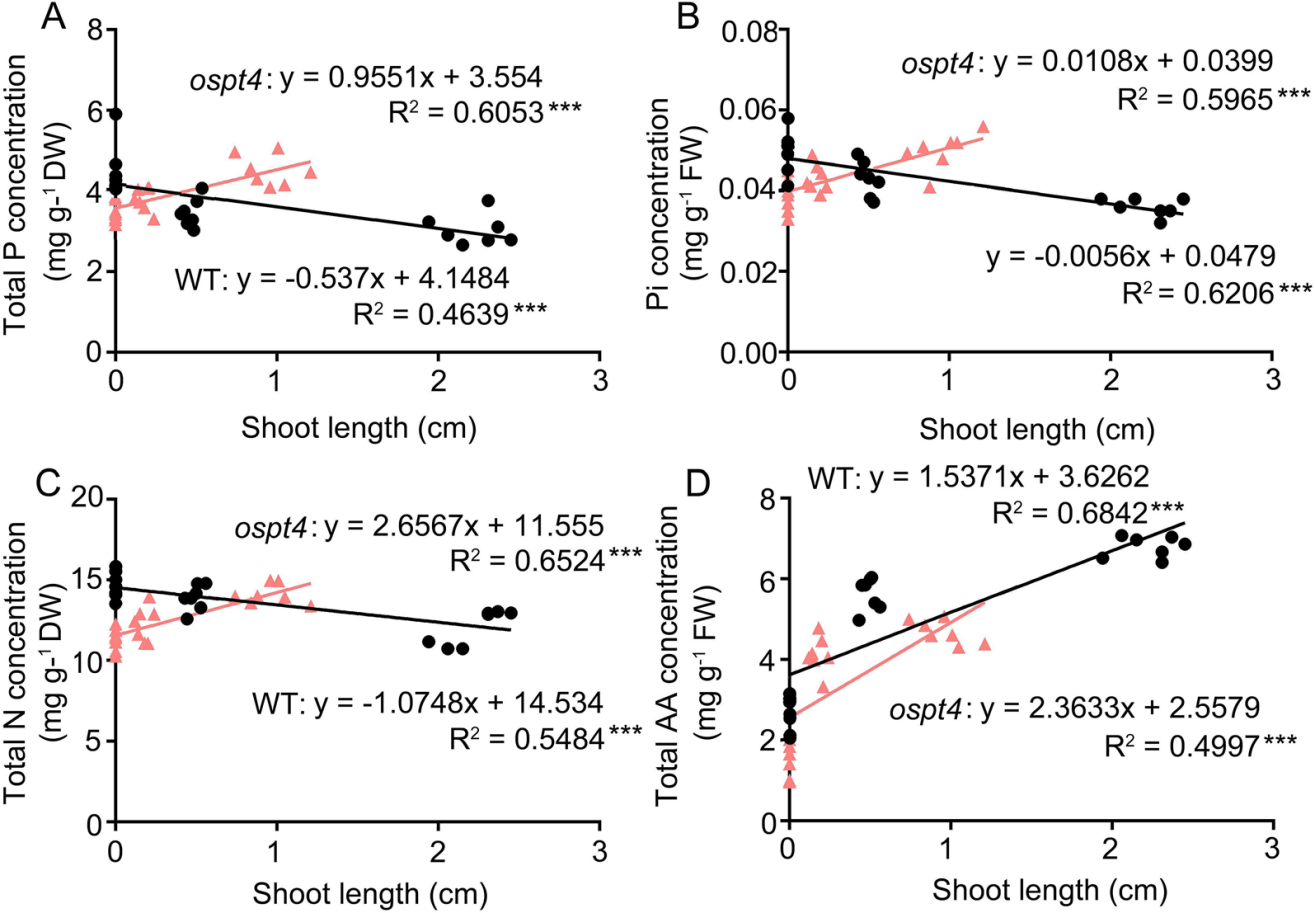
Relationships of shoot length with P and N concentration in seeds during seed germination. **A** Total P concentration in germinating seeds versus shoot length. **B** Pi concentration in germinating seeds versus shoot length. **C** Total N concentration in germinating seeds versus shoot length. **D** Total amino acid concentration versus shoot length. The black linear regression lines represent the data from WT seeds during 7 d germination, while the red linear regression lines represent the data from *OsPT4* mutants. *** indicate the significance of correlation coefficient (R) at *P* < 0.001

To investigate the effects of *OsPT4* mutation on the relationship between shoot length (the first 7 d of seed germination) and P and N accumulation in germinating seeds, total P, Pi, total N, and amino acid concentrations in germinating seeds on NP and *OsPT4* mutants were combined in correlation and regression analyses. A tight linkage was observed between shoot length and total P, Pi, total N and amino acid concentration in germinating seeds in both the NP and *OsPT4* mutant (Fig. 3A–D). In NP, shoot length was significantly negatively correlated with total P, Pi and total N. However, it was positively correlated with total P, Pi and total N in the *OsPT4* mutant (Fig. 3A–C). Moreover, shoot length was significantly positively correlated with amino acid concentration in germinating seeds in both the NP and *OsPT4* mutant (Figs. 3D), and the associated R^2^ values for these correlations were R^2^_NP_ = 0.6842 and R^2^_mutant_ = 0.4997, respectively. The slope associated with this correlation was higher in the *OsPT4* mutant than in NP (Fig. 3D). This indicates that the *OsPT4* mutation strongly affects the relationship between shoot growth during seed germination and P and N accumulation in germinating seeds. This also confirms that the *OsPT4* mutation leads to the retention of P and N nutrients in germinating seeds. In addition, the synthesis and accumulation of amino acids that are significant for seed germination are inhibited by *OsPT4* mutation.

**Fig. 4:**
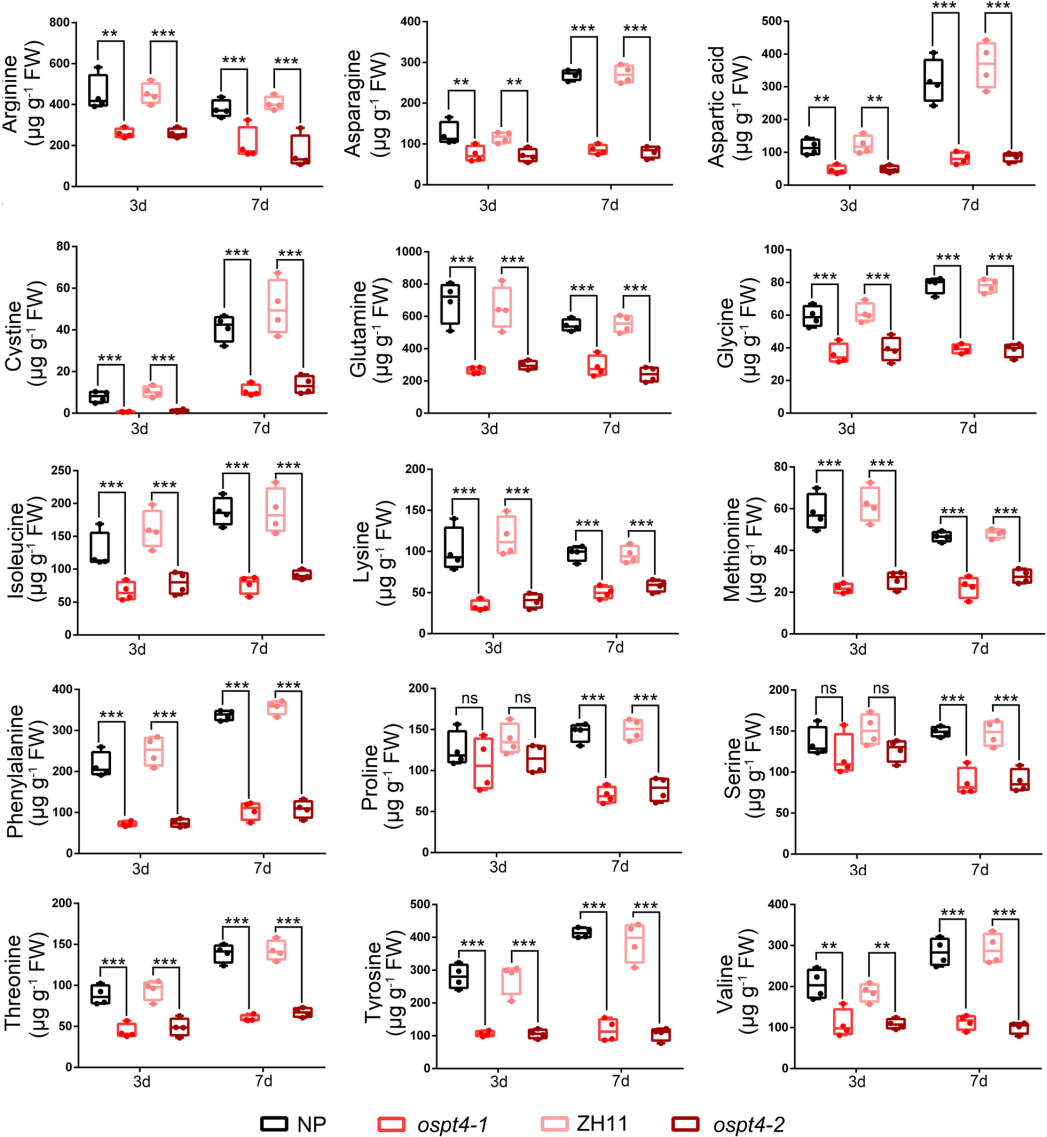
The mutation of *OsPT4* decreased the amino acid concentration in germinating seeds. Seeds of the WT and *OsPT4* mutants were grown hydroponically in nutrient rich solution. The germinating seeds were harvested for assaying the concentration of amino acid on 3 and 7 days after germination. Values are means 4 replicates, ± SE (*n* = 4). Each replicates used 10 seeds. Different letters indicates that the values differ significantly between WT and *OsPT4* mutants (**P < 0.01; ***P < 0.005; Student’s t-test). ns = not significant

Amino acid biosynthesis is necessary for seed germination (Yobi et al. 2020). In order to further understand the specific reasons for the significant decrease in N and amino acids in the germinated seeds of the *OsPT4* mutant, the amino acids were measured in germinating seeds of the WT and *OsPT4* mutants after germination for 3 and 7 days (Fig. 4). Compared with WT, the concentrations of most amino acids in the seeds of the *ospt4-1* and *ospt4-2* mutants decreased significantly. On the 3^rd^ day of germination, no significant difference was found in proline (Pro) and Ser concentrations in seeds between the WT and the mutants. However, the concentrations of arginine (Arg), asparagine (Asn), Cys, Glu, Gly, Ile, Lys, Met, Phe, Thr, Tyr and valine (Val) were all markedly decreased (Fig. 4). Among these amino acids, Glu, Phe and Tyr were the most abundant amino acids in the germinating seeds and declined by 59%, 66%, and 65% compared with the amino acids in WT (Fig. 4). In addition, the 15 amino acids (Arg, Asp, Cys, Gly, Glu, Ile, Lys, Met, Phe, Pro, Ser, Thr, Tyr and Val) listed in Fig. 4 all declined significantly in the *OsPT4* mutant seeds after 7 d of germination (Fig. 4). These results indicate that although the *OsPT4* mutation reduces the accumulation of P and N in mature seeds, it can lead to the retention of P and N during seed germination, resulting in an increase in P and N concentrations in the seeds.

To detect the transcriptomic changes and mechanism underlying the effect of *OsPT4* mutation on germinating seeds, WT and *OsPT4* mutants germinating seeds were harvested at 3 d after germination. As the determination of differentially expressed genes (DEGs) was still the core goal of the gene expression analysis, the differences in gene expression between WT and *OsPT4* mutants germinating seeds were determined by using the threshold of *P* < 0.05 and |log_2_FoldChange|> 1, as shown in Additional file 1: Figure S3. The positive and negative fold change values were the basis for the identification of up-regulated and down-regulated DEGs, respectively (Additional file 1: Figure S3A). A total of 174/168 induced and 857/832 reduced expression genes were identified as DEGs that were influenced by the mutation of *OsPT4* in germinating rice seeds (Additional file 1: Figure S3A). To further assess the functional involvement of the DEGs responding to *OsPT4* mutation in germinating seeds, the DEGs of in various metabolic pathways were mapped to the Kyoto Encyclopedia of Genes and Genomes (KEGG) database. By comparing the top 20 pathways, carbohydrate metabolism, biosynthesis of other secondary metabolites, energy metabolism, lipid metabolism, and amino acid metabolism were more enriched in DEGs in the *OsPT4* mutants germinating seeds (Additional file 1: Figure S3B, C).

**Fig. 5:**
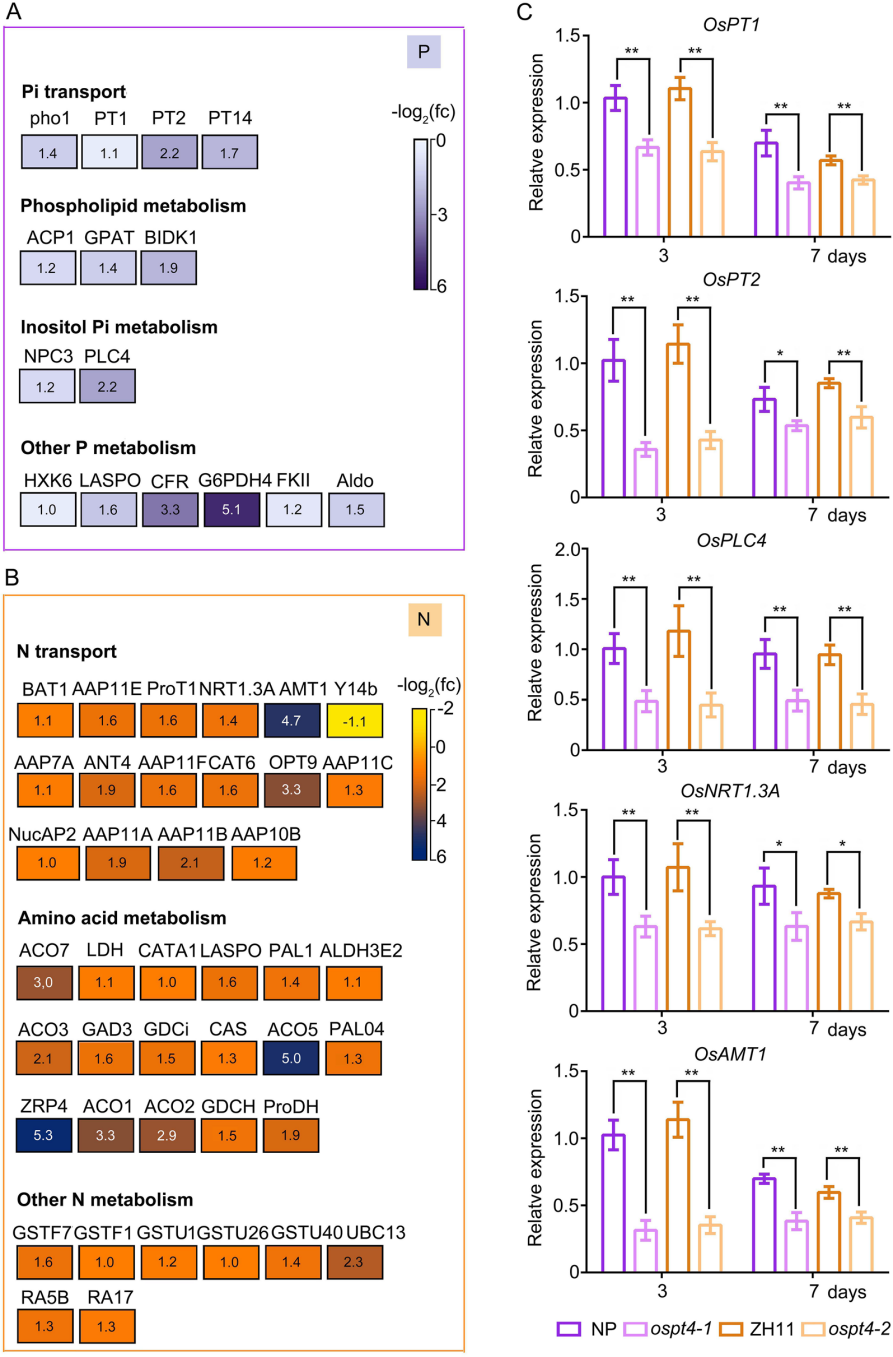
The mutation of *OsPT4* affects the expression pattern of both P and N metabolism genes. **A** and **B** The transcirptome analysis of P (**A**) and N (**B**) metabolism related genes in *OsPT4* mutants. The number inside the box represents − log_2_(fc), and the darker the color of the box, the more severe the down-regulation of gene expression in the mutant. **C** The relative expression of N and P transporter and metabolism related genes. The relative expression of *OsPT1*, *OsPT2*, *OsPLC4*, *OsNRT1.3A*, and *OsAMT1* of WT (NP and ZH11) and *OsPT4* mutants (*ospt4-1* and *ospt4-2*) seeds in 3 and 7 days after germinated. Germinating seeds were harvested for qRT-PCR analysis. *OsACTIN* (LOC_Os10g36650) were used as internal controls. Values are means 4 replicates, ± SE (*n* = 4). Each replicates used 10 seeds. and different letters on the histograms indicate that the values differ significantly (*P < 0.05; **P < 0.01; Student’s t-test)

To understand the molecular regulatory mechanism of the impact of *OsPT4* mutation on P and N homeostasis in germinating seeds, the transcriptomic changes in P and N metabolism-related genes in the germinating seeds of *OsPT4* mutants (*ospt4-1* and *ospt4-2*) were screened and analysed (Fig. 5). The abundance of 15 P metabolism- and 39 N metabolism-related genes (with relevant annotations in NCBI) showed significant up- or downregulation in both *ospt4-1* and *ospt4-2* mutant germinating seeds (Fig. 5A, B). Among the DEGs of P metabolism-related genes, 4 Pi transporters (*pho1* and *PTs*), 3 phospholipid metabolism-related genes (*ACP1*, *GPAT*, and *BIDK1*), 2 inositol Pi metabolism-related genes (*NPC3* and *PLC4*) and 6 other P metabolism-related genes were significantly downregulated by *OsPT4* mutation in germinating seeds. CFR (Os03g0267300, similar to fructose-1-6-bisphosphatase) and G6PDH4 (Os03g0412800, glucose-6-phosphate 1-dehydrogenase) were most severely affected by *OsPT4* mutation (Fig. 5A). Interestingly, *HXK6*, *LASPO*, *G6PDH4* and *Aldo* also belonged to the N metabolism-related gene set. The abundance of amino acid permease (*BAT1*) and transporter genes of nitrate (*NRT1.3A*), ammonium (*AMT1*), and amino acids (*AAPs, ProT1, CAT6 and OPT9*) was also downregulated by *OsPT4* mutation in germinating seeds, while the core subunit of the exon junction complex (*Y14b*) was upregulated by the mutation of *OsPT4* (Fig. 5B). Additionally, the abundance of many amino acid metabolism-related genes was highly reduced by *OsPT4* mutation, such as the genes encoding aminocyclopropane carboxylate oxidase (*ACOs*) and acetylserotonin O-methyltransferase 1 (*ZRP4*) (Fig. 5B). Other N metabolism-related genes were also found to be decreased in abundance by *OsPT4* mutation in germinating seeds, including the genes encoding glutathione S-transferase (*GSTFs* and *GSTUs*), ubiquitin-conjugating enzyme E2 (*UBC13*), and seed allergenic protein (RA5B and RA17). The *AMT1* (Os02g0620600), *OPT9* (Os08g0492000), *ACO5* (Os05g0149400), *ACO1* (Os09g0451000) and *ZRP4* (Os09g0344500) genes were reduced in abundance most significantly by *OsPT4* mutation in germinating seeds. We performed transcript quantification of several genes involved in P and N transport and metabolism in *OsPT4* mutant seeds after germination for 3 and 7 days (Fig. 5C). There was significant downregulation in the expression levels of *OsPT1* (Os03g0150600) and *OsPT2* (Os03g0150800), which function as Pi transporters from root to shoot, in the seeds of *OsPT4* mutants at both 3 and 7 d after germination compared with the WT seeds (Fig. 5C). Moreover, the transcript levels of *OsPLC4* (Os05g0127200) were also downregulated in *OsPT4* mutant seeds at both 3 and 7 d after germination, showing that the phenylalanine metabolism pathway was also inhibited by *OsPT4* mutation in germinating rice seeds (Fig. 5C). Similarly, the relative expression levels of the nitrate transporter gene *OsNRT1.3A* (Os02g0580900) and ammonium transporter gene *OsAMT1.2* (Os02g0620600) were downregulated by the mutation of *OsPT4*, indicating that the N transporter in germinating seeds was probably inhibited by *OsPT4* mutation (Fig. 5C). Taken together, these results suggest that the *OsPT4* mutation inhibits both P and N transport and the amino acid synthesis pathway in germinating rice seeds.

### Mutation of *OsPT4* Inhibits Seedling Growth Related to Programmed Cell Death of Aleurone Layer Reduction

**Fig. 6:**
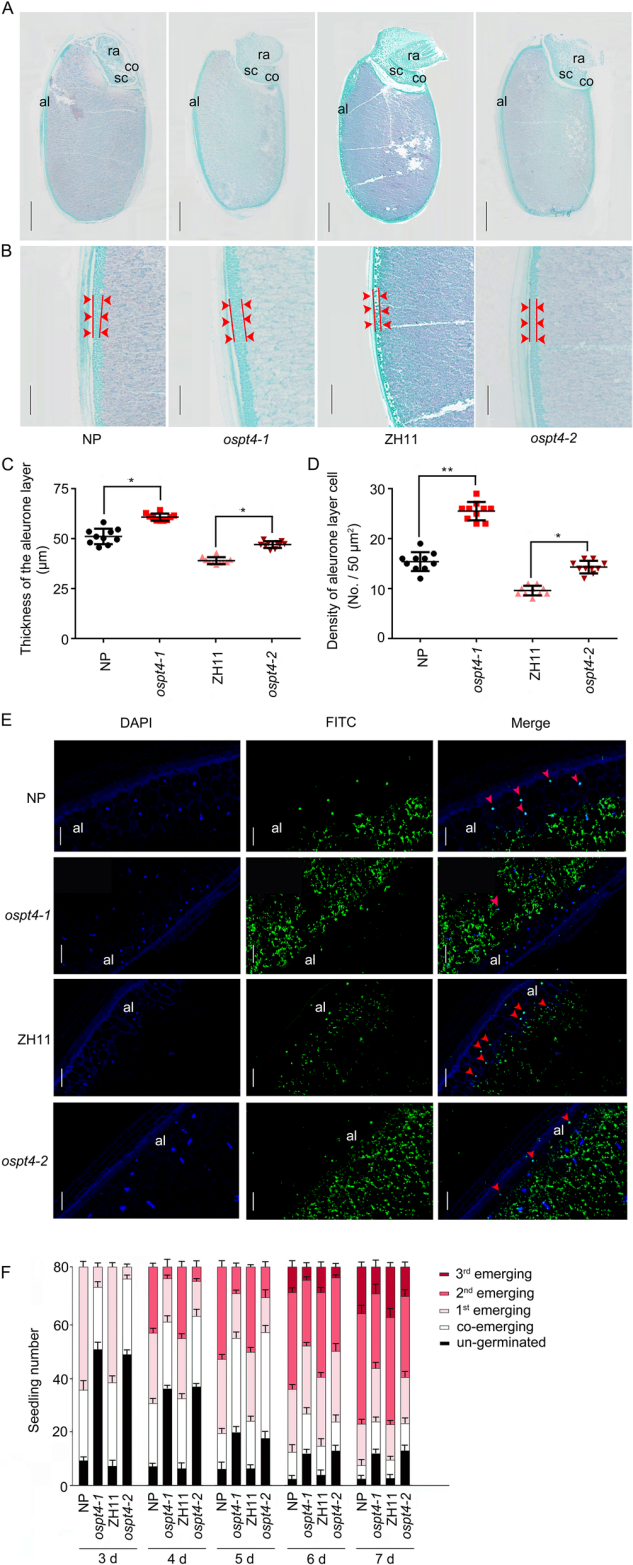
The morphology of WT and *OsPT4* mutants germinating seeds and seedlings’ leaf blade outgrowth status. **A** Longitudinal visual field of paraffin section of WT and *OsPT4* mutant seeds 3 d after germination. Bar = 1000 μm. **B** The aleurone layer of WT and *OsPT4* mutant seeds 3 d after germination. Bar = 500 μm. **C** and **D** Aleurone layer thickness (**C**) and density of aleurone layer cells (**D**). **E** Visualization of programmed cell death signals during seed germination. TUNEL assay were used to detect the nuclear DNA degradation in aleurone layer of NP and *ospt4-1* mutant. The red arrow indicates the degradation of nuclear DNA. DAPI: 4′,6-diamidino-2-phenylindole, nuclear staining (deep blue); FITC: Fluorescein isothiocyanate isomer (green); Merged: indicating the apoptotic cell (wathet blue). **F** leaf outgrowth status of WT and *OsPT4* mutant seedlings. Bar = 50 μm. Seedlings with the outgrowth of coleoptiles or different numbers of leaves were counted in (**F**) at each time point. Co, coleoptiles; 1st, 2nd, and 3rd represent the first, second, and third leaves, respectively. Values are means ± SE (*n* = 10) in (**A**–**E**) and 3 biological repeats in (F), different letters on the histograms indicate that the values differ significantly (*P < 0.05; **P < 0.01; Student’s t-test)

To investigate the effect of *OsPT4* on seed germination morphology and process, we observed the morphological changes of seeds of the WT (NP and ZH11) and *OsPT4* mutants (*ospt4-1* and *ospt4-2*) germinated for 3 d (Fig. 6A). The enlarged view of the aleurone layer of WT and *OsPT4* mutants was also examined (Fig. 6B). Combining the visual assessment with the statistical data analysis, it was found that *OsPT4* mutants displayed thicker aleurone layers with denser aleurone layer cells than the WT (Fig. 6B–D). The aleurone layer encapsulating the starch endosperm disappeared following germination, and, as was found in a previous study, this occurs through a process of programmed cell death (PCD) (Bethke et al. 1999; Fath et al. 2001). Degradation of nuclear DNA is a typical hallmark of PCD both in plant cells. Therefore, TUNEL assay was used to detect the PCD signals during seed germination (Fig. 6E). In NP germinating seeds, the degradation of nuclear DNA was found plainly in aleurone layer. However, there was few degradation nuclear DNA can be detected in the aleurone layer *ospt4-1* (Fig. 6E)*.* These results suggest that mutation of *OsPT4* disrupts the process of PCD and inhibits the disappearance of the aleurone layer. We then examined the germination of the WT and *OsPT4* mutant seeds (Fig. 6F). When grown on ½ MS medium, it took 3 days for WT seeds to reach 90% germination with 80 seeds. In contrast, it takes nearly 7 days or more for the *OsPT4* mutant to reach the same level of germination (Fig. 6F). Conformably, the outgrowth of the first three leaf blades was slower in the *OsPT4* mutants than in the WT. Approximately 20% of the WT seedlings grew 3^rd^ leaf blades on the 7^th^ day after germination, while only approximately 10% of the mutants grew 3^rd^ leaf blades (Fig. 6F). Taken together, these results show that *OsPT4* mutation inhibits PCD of the aleurone layer and leaf blade outgrowth during seed germination.

Seed germination is promoted by gibberellin (GA) in many plant species (Bewley 1997; Peng and Harberd 2002). Early studies have revealed significant chemical diversity of GAs in plants, but only GA_1_, GA_3_, GA_4_, and GA_7_ exhibit biological activities that control plant development (He et al. 2020). To further explore the reason why the *OsPT4* mutation affects seed germination, the hormone concentrations of the germinating seeds of the WT and mutants were measured (Additional file 1: Figure S4). As shown in Fig. S4, after 3 days of germination, there was no difference between the WT and *OsPT4* mutants in the concentration of GA_1_, while the concentrations of GA_3_ and GA_4_ were substantially decreased in the germinating seeds of the *OsPT4* mutants. In contrast, the concentrations of abscisic acid (ABA), jasmonic acid (JA) and jasmonic acid isoleucine (JA-Ile) were increased in the mutants compared with the WT. On the 7th day of germination, GA_1_, GA_4_, ABA, JA and JA-I were not significantly different in the *OsPT4* mutants compared with the WT. However, the concentration of GA_3_ was still significantly higher than that in the germinating seeds of the WT (Additional file 1: Figure S4). The results illustrate that the reduction of GA concentrations maybe the reason for the inhibition of seeds germination in *OsPT4* mutants.

### OsPT4 is Ubiquitinated by OsAIRP2

**Fig. 7:**
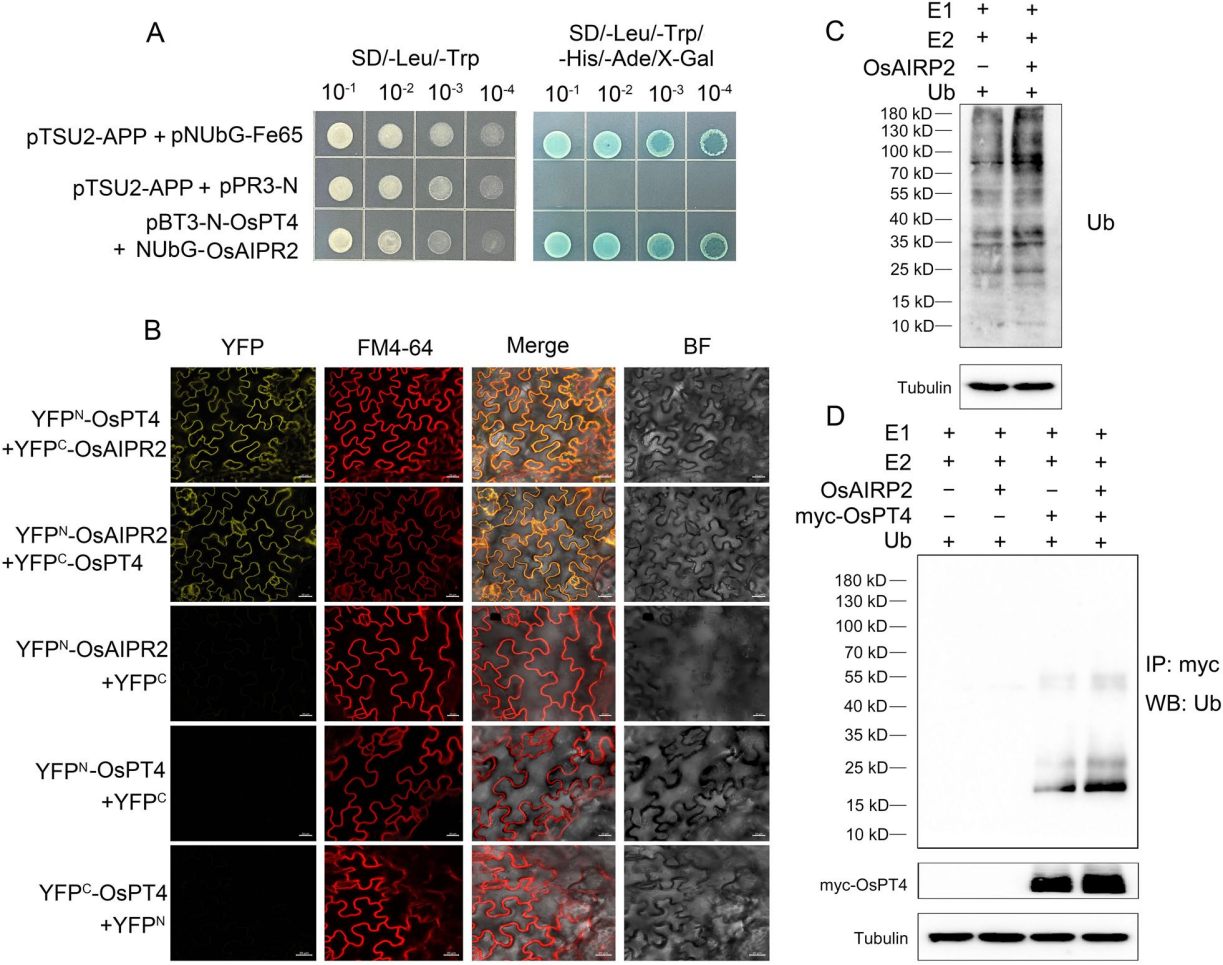
OsAIRP2 exhibits E3 ubiquitin ligase activity and ubiquitinates OsPT4. **A** OsPT4 interacts with OsAIPR2, as proved by split-ubiquitin Y2H assays. Interaction between the bait APP with Fe65 as a positive control. The coexpression of TSU2-APP and PR3-N regarded as the negative controls. **B** OsPT4 interacts with OsAIPR2 on the plasma membrane, as proved by BiFC analysis. N- and C-terminal fragments of YFP (YFP^N^ and YFP^C^) were fused to the N terminus of OsPT4 and OsAIPR2, respectively. The yellow signals indicate YFP, the red signal indicates the plasma membrane specifically stained with dye FM4-64, and the orange signals indicate the overlapping part of YFP signal and FM4-64 staining. Bars = 20 μm. NubG, the mutated N-terminal fragment of ubiquitin; X-gal, a substrate of the bacterial enzyme β-galactosidase encoded by the color reporter gene lacZ; FM4-64, BF, bright field. **C** E3 Ub ligase activity of OsAIRP2. **D** In vitro ubiquitination of OsPT4 protein. The numbers on the left indicate the molecular masses of marker proteins in kD

OsPT4 is the Pi transporter that functions in Pi absorption and translocation, playing pivotal roles in Pi utilization and seed development (Zhang et al. 2015). However, the direct binding protein of OsPT4 and its regulatory pathway in affecting nutrient accumulation and seed germination are unknown. To search for secreted proteins that bind directly to OsPT4, Y2H screening for OsPT4-interacting proteins was performed. Using OsPT4 as bait, we screened a cDNA library constructed from the mRNA of the membrane system of germinating rice seeds for interacting proteins and found 8 genes with the correct fusion in-frame encoded proteins (Additional file 2: Table S2). Os10g0445400 (*OsAIPR2*) encoded E3 ubiquitin-protein ligase (Additional file 2: Table S2), which contains 246 amino acids with a domain consisting of 38 amino acids, belonging to the RING finger protein family. AtAIRP2, a C3HC4-type RING E3 Ub ligase, is a positive regulator in the Arabidopsis ABA-dependent drought response (Cho et al. 2011). OsAIRP2 is the orthologous gene of AtAIRP2, and its function in rice has not been clearly reported. To ascertain the interacting protein of OsPT4, we tested the potential interaction between OsPT4 and OsAIPR2. Y2H assay and bimolecular fluorescence complementation (BiFC) were performed as shown in Fig. 7. The results shown in Fig. 7A suggested that the interaction of OsAIRP2 with OsPT4 in yeast was truly positive (Fig. 7A). The effects of OsAIRP2 binding on OsPT4 occur via YFP^N/C^-OsAIPR2 and YFP^N/C^-OsPT4 coexpression in tobacco leaves. Transfected leaves were then observed using laser confocal microscopy, and a strong YFP fluorescence signal was observed in the cell membrane coexpressing YFP^N/C^-OsPT4 and YFP^N/C^-OsAIPR2 (Fig. 7B). We further performed in *vitro* ubiquitination assays using a Arabidopsis E1 (UBA1) and E2 (UBC8), and OsAIRP2 proteins. When OsAIRP2 was added to the reaction, a protein ladder was detected using anti-Ub antibody, the overall grayscale of the lane 2 increased. This infers that OsAIRP2 has E3 Ub ligase activity (Fig. 7C). We further tested whether OsPT4 is a ubiquitination substrate of OsAIRP2. Incubation of myc-OsPT4 with E1, E2, and SDEL1 led to the detection of a protein ladder signal (Fig. 7D, lane 4), whereas incubation with E1 and E2 alone did not result in the addition of ubiquitin to OsPT4 (Fig. 7D, lane 3). These results indicates that OsAIRP2 is a functional E3 Ub ligase able to ubiquitinate OsPT4 in *vitro*.

## Discussion

The 13 members of the PHT1 family are the major contributors to Pi uptake and translocation and play different roles in rice growth and development. Among these genes, the relative expression of *OsPT1*, *OsPT4* and *OsPT8* is much higher than that of the others during seed development (Zhang et al. 2015). Seed germination is the key process in ensuring the continuation of plant life. The process of germination begins with the imbibition of water and ends with the protrusion of the coleoptile and radicle (Bewley 1997). Rice seed germination is divided into the following three processes: Step I, rapid water uptake and DNA repair (0–24 h after germination) (Macovei et al. 2011); step II, mitochondrion synthesis (24–48 h after germination) (Howell et al. 2006); and step III, exiting of embryonic axes from the structures surrounding the embryo (48–72 h after germination) (Bewley 1997; Yang 2007). The relative expression of *OsPT1* and *OsPT8* was higher than that of the other genes during all 3 steps of the germination process. It seems that both genes play more important roles in the exposure of embryonic axes because their relative expression has a very obvious upward trend during step III (Fig. 1). Interestingly, the relative expression pattern of *OsPT4* was quite different from that of *OsPT1* and *OsPT8*. No expression was found in the process of rapid water uptake during step I, while an upward trend was detected in steps II and III (Fig. 1). A previous study showed that numerous larger starch granules accumulate around the vascular tissues, scutella, and epithelium during step II, perhaps making step II a critical stage for rice seed germination (Matsukura et al. 2000). In addition, *OsPT4* was sensibly up-regulated in low Pi seeds comparing to normal seeds (Additional file 1: Figure S1). This suggests that *OsPT4* may play a pivotal role in nutrient accumulation to prepare sprouting protuberances and in the process of sprouting protuberances in low Pi seeds. The expression patterns of *OsPT6* and *OsPT7*, which play dual roles in P accumulation in anthers and Pi translocation from roots to shoots, were similar to the *OsPT4* expression pattern (Ai et al. 2009; Dai et al. 2022). This result indicated that *OsPT6* and *OsPT7* also likely functioned in the Pi distribution and transportation of germinating seeds during the embryonic axis exposure stage. The relative expression of *OsPT9* and *OsPT10* was analogous—abundant during step I of germination (Fig. 1)—confirming the redundant function of these genes in Pi uptake (Wang et al. 2014). OsPT3 mediates Pi uptake, translocation, and remobilization, interacting with OsPT2 (Chang et al. 2019). The relative expression of *OsPT3* was extremely unique, reaching a peak value after the rapid water uptake of seeds (Fig. 1). This suggests that *OsPT3* may mainly have a role in the imbibition process. These results seem to indicate that members of the Pht1 family interact and participate in the regulation of nutrient distribution and metabolic mechanisms in the process of rice seed germination. Therefore, it will be very interesting to further explore how the Pht1 family members divide the work in nutrient transportation and distribution in germinating seeds.

N and P are the two most abundant mineral nutrients for plants, and their coordinated acquisition is vital for plants to achieve nutritional balance and optimal growth under a fluctuating nutritional environment (Güsewell 2004; Khan et al. 2014; Luo et al. 2016). The interaction between N and P consists of two parts: N regulated P reaction and P regulated N reaction. Recent studies have well described the pre response of N regulation, while the N response of P regulation is still largely unclear (Hu and Chu 2020). It confirmed that crops have interactive effects on N and P uptake and utilization (Güsewell 2005). Additionally, Pi starvation was associated with amino acid accumulation in Arabidopsis, indicating the potential impact on amino acid metabolism of Pi transportation and mobilization (Aleksza et al. 2017). In our study, we elucidated the mechanism of partial N response to P accumulation and transportation. Total P, Pi and total N retention occurred in the germinating seeds of *OsPT4* mutants (Fig. 2). The correlation and regression analyses of shoot growth conditions and P/N concentrations in germinating seeds also illustrated that *OsPT4* mutation leads to the retention of P and N in germinating seeds, while the P and N nutrients in WT seeds gradually decrease with the passage of germination time (Fig. 3). OsPT4 was shown to be a high-affinity Pi transporter that affects Pi uptake, translocation from roots to shoots, and Pi accumulation in the embryos of seeds (Zhang et al. 2015). We speculate that OsPT4 may act as a Pi transporter responsible for the redistribution of Pi from seeds to other vegetative organs during seed germination. Similarly, *OsPT8*, which has functional redundancy with *OsPT4* during seed development, is responsible for the redistribution of Pi from source to sink organs (Zhang et al. 2015; Li et al. 2015). In addition, the relative expression of *OsPT1* and *OsPT2* was down-regulated by *OsPT4* mutation in germinating seeds (Fig. 5C). Notably, the relative expression of other Pi transporter genes was mostly up-regulated by *OsPT4* mutation in developing seeds in our previous study (Zhang et al. 2015). Further research is needed to detect whether this divergence in the expression pattern of Pi transporter genes indicates that *OsPT4* plays a unique role in Pi distribution to growing vegetative tissues during seed germination, which is different from the process of seed development. In addition, the relative expression of *OsNRT1.3A* and *OsAMT1.2,* which encode nitrate and ammonium transporters, was also down-regulated by *OsPT4* mutation in germinating seeds (Fig. 5C). Therefore, P and N retention in germinating seeds of *OsPT4* mutants may be caused by the disruption of Pi, nitrate and ammonium transport function from germinating seeds to other vegetative tissues in *OsPT4* mutant seeds. This result indicates the synergistic transport of P and N during seed germination. When the accumulation and transportaion of Pi are hindered, N translocation is also inhibited to the same extent.

Amino acids are the core of glycolysis, the pentose Pi pathway and the tricarboxylic acid (TCA) cycle are an important energy source when carbohydrates are scarce. Along with the process of seed germination, many amino acid forms are also converted, which also means that amino acid metabolism is significant in seed germination (Rios-Iribe et al. 2011). As a consequence, the alteration in amino acid concentration is most noteworthy (Figs. 2 and 4). In germinating seeds, the reduction in amino acid concentration by the *OsPT4* mutation was always present. Furthermore, the reduction increased with the passage of germination time (Figs. 2D and 4D). Based on the transcriptome results, several amino acid transporter genes and amino acid metabolism-related genes were down-regulated in germinating seeds (Fig. 5B). While as we discussed above, *OsPT4* mutation decreased the relative expression of the ammonium and nitrate transporter genes (Fig. 5C), which indicates that *OsPT4* mutation may not only inhibit the translocation of N from seeds to shoots, but also suppress the synthesis of amino acids in germinating seeds. It is known that proteases break down the storage proteins to provide free amino acids and small peptides for contributing to the synthesis of structural and functional proteins of the developing radicle. Therefore, the free amino acid in germinating seeds can increase markedly in concentration (Fait et al. 2006). Our study also revealed that after 7 d of germination, Arg, Asp, Cys, Phe and Thr in the seeds still showed a significant increase compared with the seeds after 3 d of germination. Among the different types of amino acids, the Asp family pathway plays a crucial role during energy shortage (Credali et al. 2013). In cotton (*Gossypium hirsutum*), the flow of N from Arg to Asp during the first 3 days of germination has been preliminarily detected (Dilworth and Dure 1978). Asp can be converted by the Asp aminotransferase to Glu, an important precursor to produce Asp (Gaufichon et al. 2016). Inhibition of the Val pathway also leads to embryo lethality or poorly developed seeds with reduced germination rates (Gipson et al. 2017). Moreover, Ile, Lys and Phe can facilitate seed germination (Rios-Iribe et al. 2011). This indicates that the *OsPT4* mutation has an inhibitory effect on the synthesis of amino acids in seeds during germination, and may also inhibit seed germination as a result.This also illustrates that *OsPT4* affects amino acid transportation and metabolism in germinating seeds. Taken together, these results indicate that *OsPT4* has a synergistic effect on the consumption and assimilation of P and N in seeds during the germination process.

The aleurone layer is a part of the endosperm. In the germinated rice seeds, dying cells were first observed in the aleurone layer neighbouring the scutellum. With the increase in the number of dead cells, the affected areas further spread to other areas of seeds (Mrva et al. 2006; Domínguez et al. 2014). Ultimately, the aleurone layer and scutellum disappear by PCD after germination (Bethke et al. 1999; Fath et al. 2001). In this study, the mutation of *OsPT4* resulted in a more complete and compact scutellum and aleurone layer in germinating seeds (Fig. 6). Moreover, the *OsPT4* mutation caused a serious reduction in GA_3_ and GA_4_ concentrations and an increase in ABA concentrations in the germinating seeds (Additional file 1: Figure S4). Specifically, GAs are involved in breaking dormancy by stimulating the secretion of hydrolytic enzymes, thereby weakening barrier tissues such as the seed coat. They promote seed germination, while ABA preserves seed dormancy (Bethke et al. 1999; Domínguez et al. 2004). GAs stimulate the onset of PCD in the aleurone layer (Wang et al. 1996; Bethke et al. 1999; Kuo et al., 1996). In contrast, ABA-treated aleurone protoplasts remain viable, and PCD is postponed (Fath et al. 2000). In addition, JA and its derivatives, which were also reduced significantly in germinating seeds of the *OsPT4* mutant, have been found to inhibit seed germination by disturbing the peroxisomal adenosine triphosphate (ATP) binding cassette transporter or core b-oxidation process (Dave et al. 2011; Liu et al. 2015; Wang et al. 2020). These studies show that *OsPT4* alters hormone metabolism and accumulation in germinating seeds, causing PCD delayed in the aleurone layer.

AtAIRP2, a C3HC4-type RING E3 Ub ligase, is a positive regulator in the Arabidopsis ABA-dependent drought response (Cho et al. 2011). Mutation of *AtAIRP2* reduced the sensitivity of Arabidopsis seed germination to ABA, resulting in a higher germination rate of the mutant under different ABA treatments (Oh et al. 2017). OsAIRP2 is the orthologous gene of AtAIRP2, and its function in rice has not been clearly reported. The results in Fig. 7 indicate that OsAIRP2 may target OsPT4 via ubiquitination to regulate the degradation of OsPT4. This finding also indicates that OsAIRP2 may target OsPT4 via ubiquitinating to regulate the degradation of OsPT4. Interestingly, the ABA concentration in *ospt4* mutants was increased significantly (Additional file 1: Figure S4). It is possible that ubiquitination of OsPT4 by OsAIRP2 is one of the regulatory pathways for the accumulation of ABA in germinating seeds.

## Conclusions

OsPT4 is a high-affinity Pi transporter that has a positive impact on Pi uptake and seed germination. The mutation of *OsPT4* caused P and N retention and a continuous reduction in multiple amino acid concentrations in germinating seeds. We conducted a preliminary study on the molecular regulatory mechanism of *OsPT4* regulating seed germination and found that, *OsPT4* mutation inhibits the expression of genes related to P and N transportation and amino acid synthesis in germinating seeds. Moreover, OsPT4 was ubiquitinated by OsAIRP2 in vitro, which is a C3HC4-type RING E3 Ub ligase in rice. Furthermore, *OsPT4* mutation causes PCD delayed in the aleurone layer and inhibits leaf outgrowth during germination process. In the future, synchrotron radiation and isotope experiments can be used to further understand the impact of *OsPT4* on the P and N transportation dynamics during rice seed germination. Moreover, the creation of *OsAIRP2* transgenic rice materials can help further explore the ubiquitination regulation mechanism of *OsPT4* and the molecular mechanism by which this mechanism affects the P homeostasis in rice.

### Supplementary Information

**Additional file 1**. **Figure S1**: The relative expression of Pht1 family members in germinating rice seeds. **Figure S2**: Molecular analysis of OsPT4 mutants. **Figure S3**: Transcriptome analysis of WT and ospt4 germinating seeds. **Figure S4**: The mutation of OsPT4 altered the hormone concentration in germinating seeds.

**Additional file 2**. **Table S1**: Primers used for the identification of OsPT4 mutants, qRT-PCR and vector construction. **Table S2**: Proteins prediction that may interact with OsPT4 by yeast two-hybrid sieve library.

## Data Availability

All data supporting the findings of this study are available from the corresponding author on reasonable request.

## Abbreviations

PT/PHT: Phosphate transporter
PCD: Programmed cell death
P: Phosphorus
Pi: Phosphate
N: Pitrogen
GA: Gibberellin
Met: Methionine
Leu: Leucine
Ile: Isoleucine
Lys: Lysine
Phe: Phenylalanine
Trp: Tryptophan
Tyr: Tyrosine
Thr: Threonine
Asp: Aspartate
Ser: Serine
Gly: Glycine
PHR1: Phosphate starvation response 1
PSR: Phosphate starvation response
ER: Endoplasmic reticulum
PHF1: Phosphate transporter traffic
Ub: Ubiquitin
Lys: Lysine
DEGs: Differentially expressed genes
GO: Gene ontology
KEGG: Kyoto Encyclopedia of Genes and Genomes
Cys: Cystine
Pro: Proline
Arg: Arginine
Asn: Asparagine
Val: Valine
ABA: Abscisic acid
JA: Jasmonic acid
JA-Ile: Jasmonic acid isoleucine
