# Correction: Phosphate Transporter OsPT4, Ubiquitinated by E3 Ligase OsAIRP2, Plays a Crucial Role in Phosphorus and Nitrogen Translocation and Consumption in Germinating Seed

**DOI:** 10.1186/s12284-024-00689-w

**Published:** 2024-02-03

**Authors:** Yafei Sun, Fang Zhang, Jia Wei, Ke Song, Lijuan Sun, Yang Yang, Qin Qin, Shiyan Yang, Zhouwen Li, Guohua Xu, Shubin Sun, Yong Xue

**Affiliations:** 1 Institute of Eco-Environment and Plant Protection, Shanghai Academy of Agricultural Sciences, 201403 Shanghai, China; 2 State Key Laboratory of Crop Genetics and Germplasm Enhancement, Key Laboratory of Plant Nutrition and Fertilization in Low-Middle Reaches of the Yangtze River, Ministry of Agriculture, Nanjing Agricultural University, 210095 Nanjing, China; 3 Shanghai Key Laboratory of Protected Horticultural Technology, Shanghai Academy of Agricultural Sciences, 201403 Shanghai, China; 4 Key Laboratory of Crop Physiology, Ecology and Genetic Breeding Ministry of Education, Jiangxi Key Laboratory of Crop Physiology, Ecology and Genetic Breeding, Jiangxi Agricultural University, 330045 Nanchang, China; 5 College of Fisheries and Life Science, Shanghai Ocean University, 201306 Shanghai, China; 6 Jilin Provincial Key Laboratory of Agricultural Biotechnology, Jilin Academy of Agricultural Sciences, 130033 Changchun, China

**Correction: Rice (2023) 16:54**

10.1186/s12284-023-00666-9

The Acknowledgements section was incorrectly published in the original article [1] and should have read as below:

This work was supported by Shanghai Agriculture Applied Technology Development Program, China (Grant No. 2022-02-08-00-12-F01202), Natural Science Foundation of Shanghai (23ZR1469200), the Chinese National Natural Science Foundation (31902102), Natural Science Foundation of Shanghai (21ZR1443300), Sponsored by Shanghai Rising-Star Program (23QB1405900), Flagship Project of Eco-Environmental Protection Research Institute, SAAS (SK-JC 2023-1), NAES035AE03 National Agricultural Experimental Station for Agricultural Environment, Fengxian (grant number: NAES035AE03).

The original article has been corrected.
