# Supplementary material for: Phosphate Transporter OsPT4, Ubiquitinated by E3 Ligase OsAIRP2, Plays a Crucial Role in Phosphorus and Nitrogen Translocation and Consumption in Germinating Seed
Source: Rice (N Y). 2023 Dec 6;16:54. doi: 10.1186/s12284-023-00666-9 (PMC10697913; doi:10.1186/s12284-023-00666-9)
Supplement: Supplementary file 1 — Additional file 1. Figure S1: The relative expression of Pht1 family members in germinating rice seeds. Figure S2: Molecular analysis of OsPT4 mutants. Figure S3: Transcriptome analysis of WT and ospt4 germinating seeds. Figure S4: The mutation of OsPT4 altered the hormone concentration in germinating seeds. [file 12284_2023_666_MOESM1_ESM.doc]

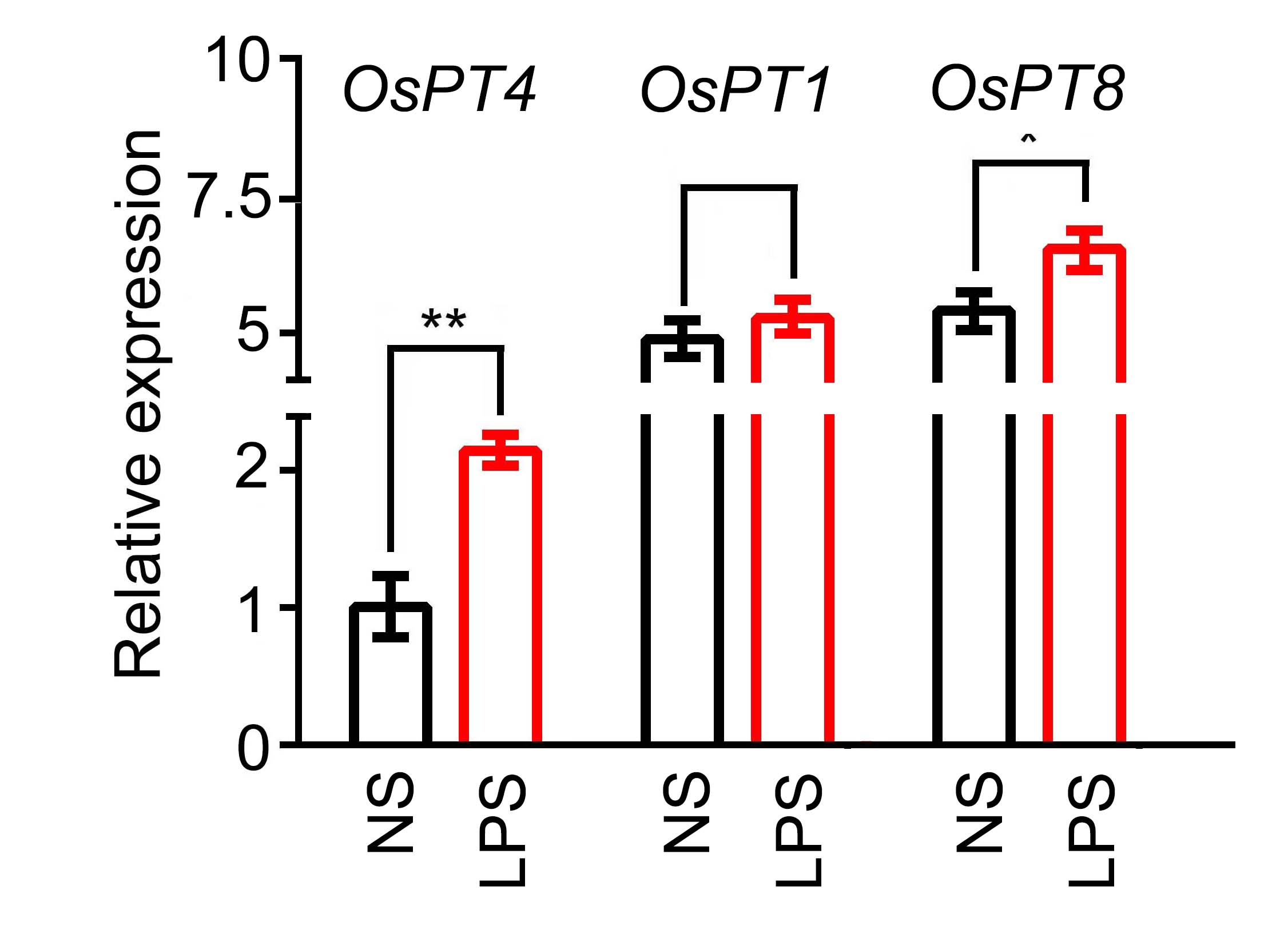


**Figure S1.** The relative expression of *OsPT1*, *OsPT4* and *OsPT8* in the normal and low Pi seeds during seed germination. NP seeds harvested under normal (NS) and low Pi (LPS) soil condition. The soil Pi concentration in normal and low Pi treatment was 37.3 and 9.53 mg/kg, respectively. Seeds were germinated for 3 d and harvested for qRT-PCR assay to detect the relative expression of *OsPT1*, *OsPT4* and *OsPT8*. *OsACTIN* (LOC_Os10g36650) were used as internal controls. Values are means ±SE (*n* = 3). Different letters on the histograms indicate that the values differ significantly (**P* < 0.05; ***P* < 0.01; Student’s t-test).


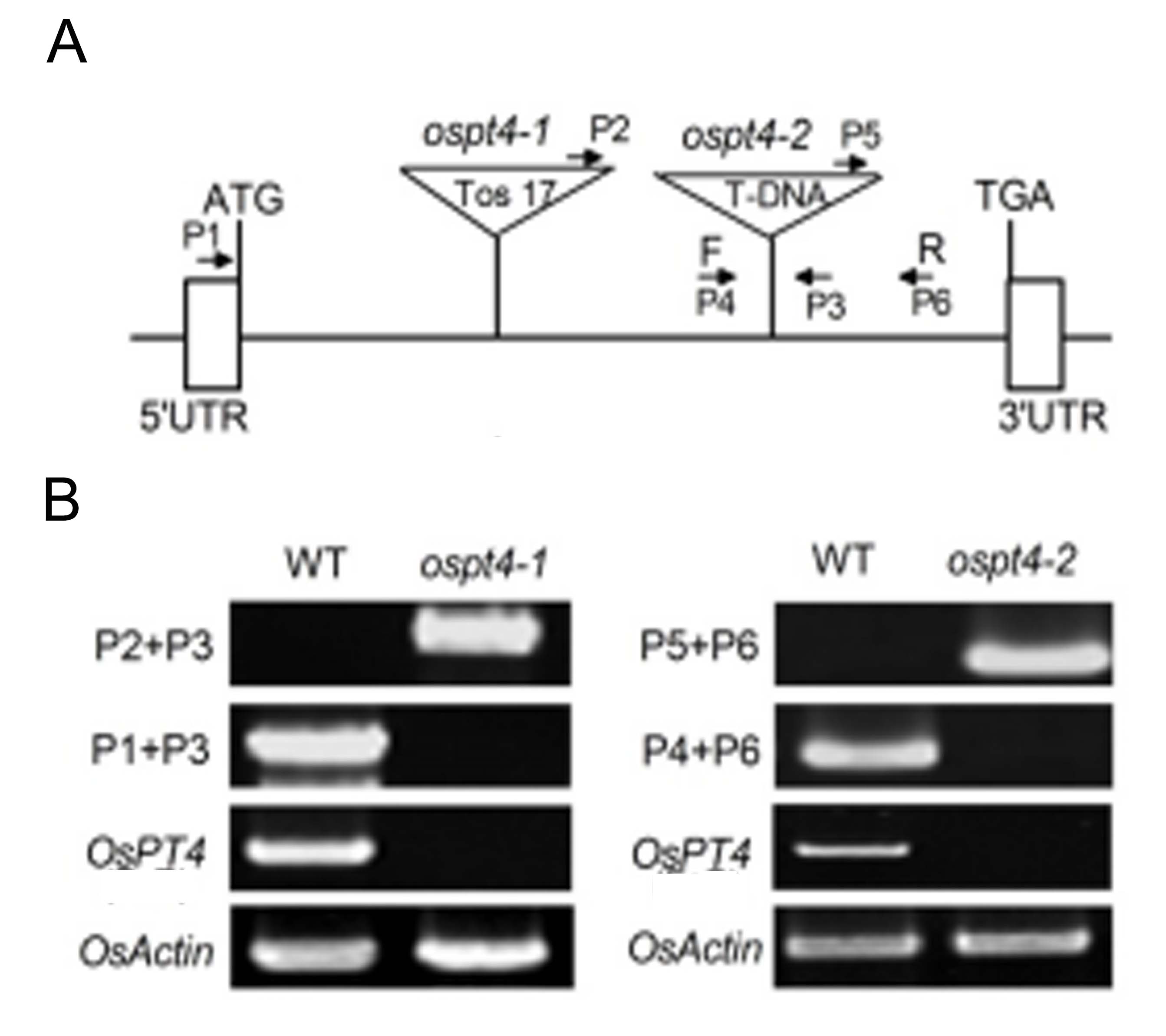


**Figure S2.** Molecular analysis of *OsPT4* mutants. (A) Schematic representation of the genomic organization of *OsPT4*, positions of Tos 17, and T-DNA insertion sites, and primer sets used for genotyping (P1 to P6), and RT-PCR analysis (F and R). (B) Two rounds of PCR and RT-PCR analysis for identification of homozygous *OsPT4* mutants and detecting relative expression of *OsPT4* in leaf blades of WT, *ospt4-1*, and *ospt4-2* plants.


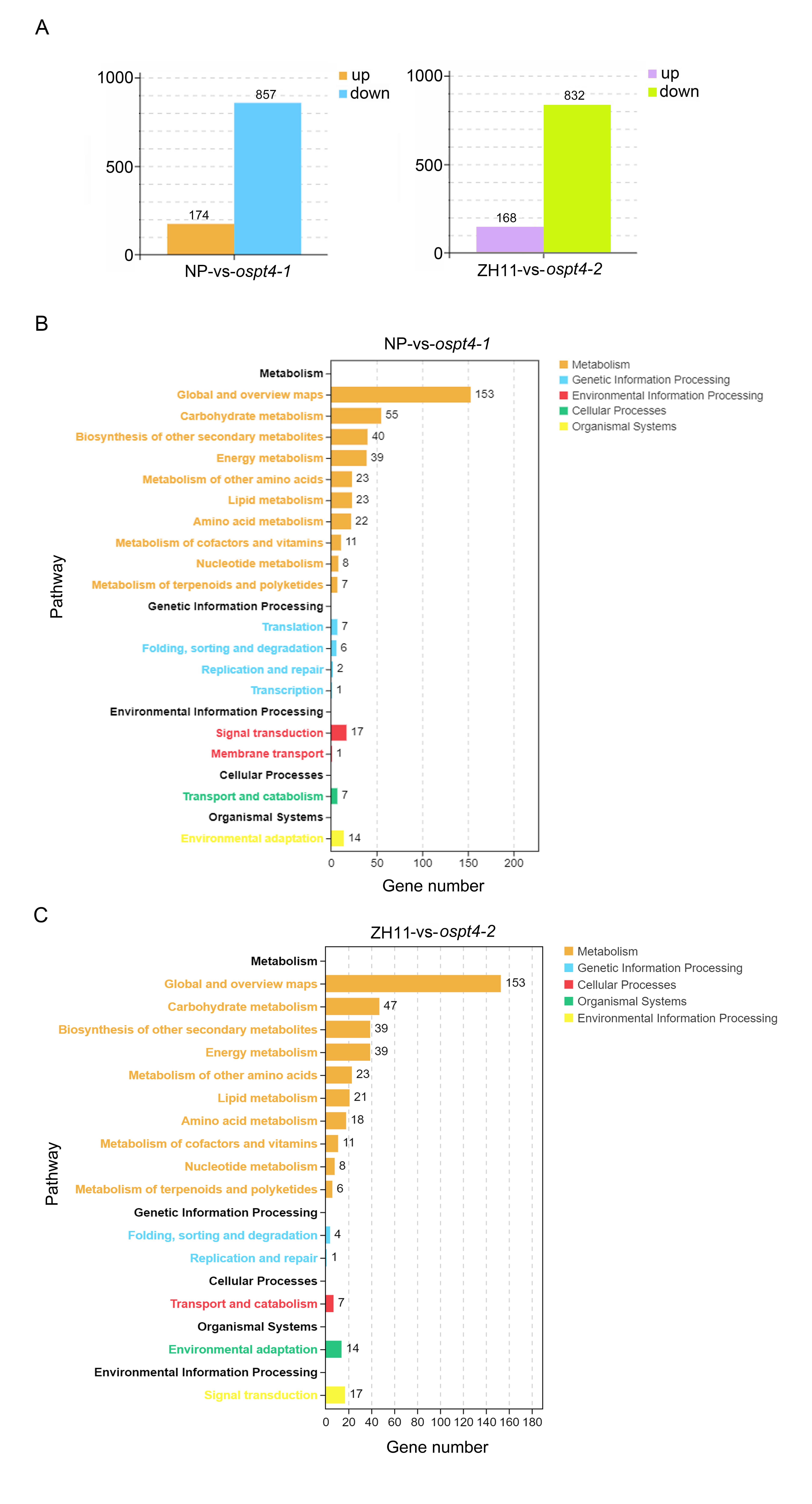


**Figure S3.** Transcriptome analysis of WT and *ospt4* germinating seeds. (A) Differential gene expression of WT (NP and ZH11) and *ospt4* (*ospt4-1* and *ospt4-2*) germinating seeds. Volcano plot and Histogram showing the (log_2_FC, −log_10_FDR) expression of the deferentially expressed genes (DEGs, *P* < 0.05). Number of DEGs, either up-(orange) or down-regulated (blue) in *ospt4-1* germinating seeds. (B and C) Kyoto Encyclopedia of Genes and Genomes pathway enrichment analysis of DEG in *ospt4-1* (B) and *ospt4-2* (C) germinating seeds.


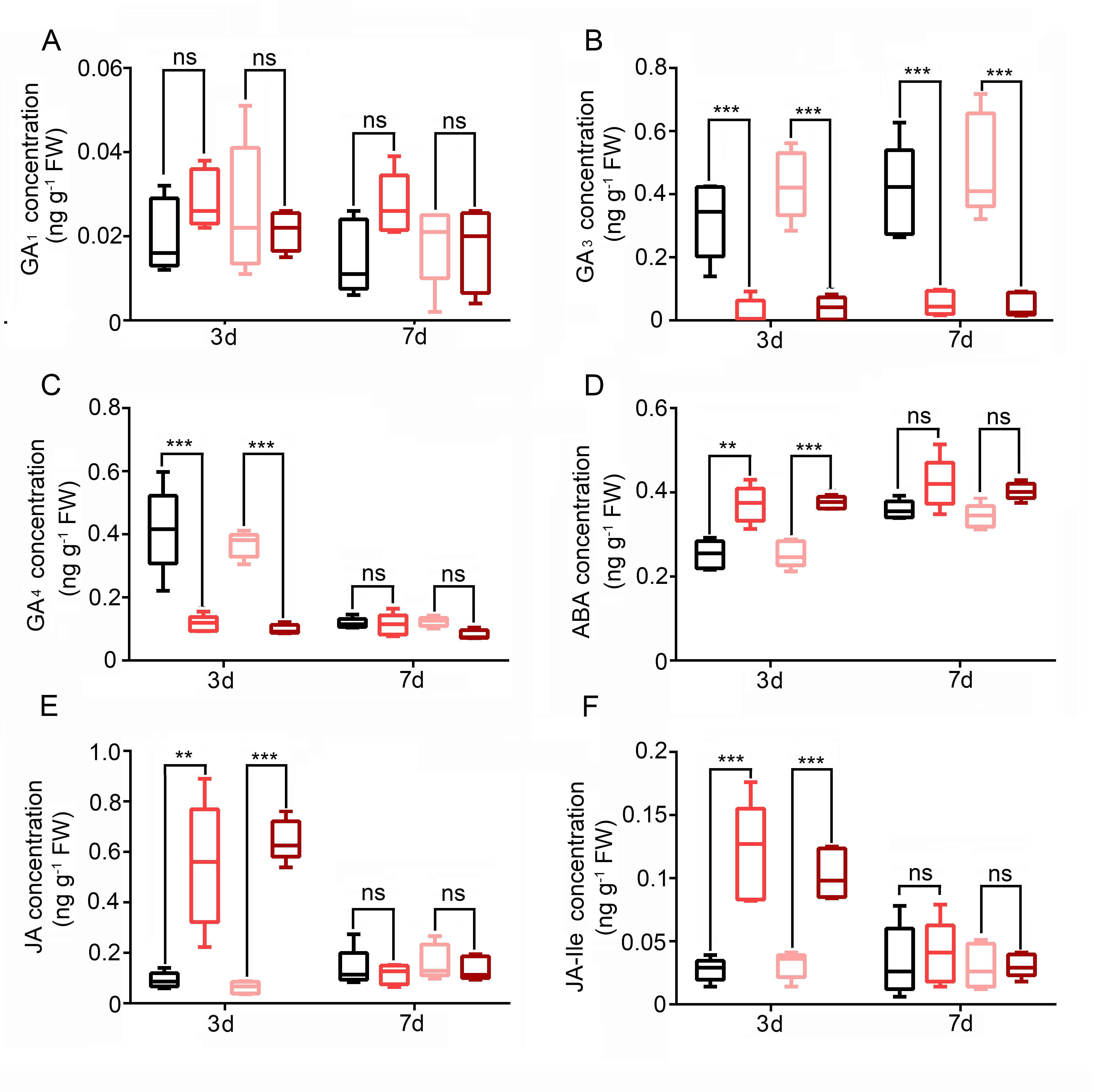


**Figure S4.** The mutation of *OsPT4* altered the hormone concentration in germinating seeds. Seeds of the WT and *OsPT4* mutants were grown hydroponically in nutrient rich solution. The germinating seeds were harvested for assaying the concentration of amino acid on 3 and 7 days after germination. ABA: abscisic acid; JA: Jasmonic acid; JA-Ile: Jasmonic acid-Ile. Values are means ±SE (*n* = 4). Different letters indicates that the values differ significantly between WT and *OsPT4* mutants (***P* < 0.01; ****P* < 0.005; Student’s t-test). ns = not significant.
