# Supplementary material for: Phosphate Transporter OsPT4, Ubiquitinated by E3 Ligase OsAIRP2, Plays a Crucial Role in Phosphorus and Nitrogen Translocation and Consumption in Germinating Seed
Source: Rice (N Y). 2023 Dec 6;16:54. doi: 10.1186/s12284-023-00666-9 (PMC10697913; doi:10.1186/s12284-023-00666-9)
Supplement: Supplementary file 2 — Additional file 2. Table S1: Primers used for the identification of OsPT4 mutants, qRT-PCR and vector construction. Table S2: Proteins prediction that may interact with OsPT4 by yeast two-hybrid sieve library. [file 12284_2023_666_MOESM2_ESM.doc]

Table S1.Primers used for the identification of *OsPT4* mutants, qRT-PCR and vector construction.

| **S.No.** | **Purpose** | **Primer name** | **Primer sequence** |
| --- | --- | --- | --- |
| 1 | Identification of *OsPT4* mutants | *OsActin-*RT-FP | GGAACTGGTATGGTCAAGGC |
|  |  | *OsActin-*RT-RP | AGTCTCATGGATAACCGCAG |
|  |  | *OsPT4-*RT-FP | atcgtggaggagcaggagaagg |
|  |  | *OsPT4-*RT-RP | catcgtcatcgtcctcgttctc |
|  |  | P1 | ATCTTCTCGTAGCCACGTGC |
|  |  | P2 | GAGAGCATCATCGGTTACATCTTCTC |
|  |  | P3 | CCTTCTGGAACAGGTTCGAC |
|  |  | P4 | atcgtggaggagcaggagaagg |
|  |  | P5 | TTCGGGGGGATCATGGACGTACCAG |
|  |  | P6 | catcgtcatcgtcctcgttctc |
| 2 | Primers for qRT-PCR | *OsPT1*-qPCR-FP | CGCTTCCGTACGAGTGGTAGT |
|  |  | *OsPT1*-qPCR-RP | GGTTCTTTCAAATCCAGGGAAA |
|  |  | *OsPT2*-qPCR-FP | GACGAGACCGCCCAAGAAG |
|  |  | *OsPT2*-qPCR-RP | TTTTCAGTCACTCACGTCGAGAC |
|  |  | *OsPT3*-qPCR-FP | TGCGACTGCTGATTCAGTACGT |
|  |  | *OsPT3*-qPCR-RP | ACAAATGCCATCAAATATGAACAGA |
|  |  | *OsPT4*-qPCR-FP | TTCTGCTAGTGTACCAAACAAAATTACA |
|  |  | *OsPT4*-qPCR-RP | CTAAGTGGCATTTATAATATCAACAGTAACC |
|  |  | *OsPT6*-qPCR-FP | CCGCCCCTGCAAACTGTA |
|  |  | *OsPT6*-qPCR-RP | CAACTGGCGGTTTCTTCGAT |
|  |  | *OsPT7*-qPCR-FP | GCAAGTCGCTCGAGGAGATG |
|  |  | *OsPT7*-qPCR-RP | TGGAATTAACGGGTGGATCAC |
|  |  | *OsPT8*-qPCR-FP | AGAAGGCAAAAGAAATGTGTGTTAAAT |
|  |  | *OsPT8*-qPCR-RP | AAAATGTATTCGTGCCAAATTGCT |
|  |  | *OsPT9*-qPCR-FP | AGAAAAACATAGGCTTGTCATCCTTT |
|  |  | *OsPT9*-qPCR-RP | AAAACCTAAGAAGCACTGTAAATAAATCC |
|  |  | *OsPT10*-qPCR-FP | ATGTCGCCCATCCTTCCA |
|  |  | *OsPT10*-qPCR-RP | TCGCTTTCCGACGATGATC |
|  |  | *OsPT12*-qPCR-FP | AACGAGATGACGAACACTTGCA |
|  |  | *OsPT12*-qPCR-RP | TCCTGTACCTAAAAAGCAAGTACTAACATAGT |
|  |  | *OsActin*-qPCR-FP | GAACTGGTATGGTCAAGGCTG |
|  |  | *OsActin*-qPCR-RP | ACACGGAGCTCGTTGTAGAAG |

| 3 | Primers for BiFC and Y2H construct vectors | pCAMBIA1300-35S-*OsPT4*-YFPc-FP | TTCAGTCGATCTGATCAAGAGACAGGATCCATGGCCGGCGAGCTCAAGGTGCTGA |
| --- | --- | --- | --- |
|  |  | pCAMBIA1300-35S-*OsPT4*-YFPc-RP | TCTGCTTGTCCATCGGTGCACTAGTGTCGACAGCTGGCGGCGCCGGCGCAGTCCGG |
|  |  | pCAMBIA1300-35S-*OsPT4*-nYFP-FP | CGAGAGTCGATCTGATCAAAGAGACAGGATCCATGGCCGGCGAGCTCAAGGTGCTGA |
|  |  | pCAMBIA1300-35S-*OsPT4*-nYFP-RP | CGAGCTCCTACATCGGTGCACTAGTGTCGACTCAAGCTGGCGGCGCCGGCGCAGTC |
|  |  | pCAMBIA1300-35S-*OsAIRP2*-YFPc-FP | TTACAGTCGATCTGATCAAGAGACAGGATCCATGCGGAGGAGGTTCCAGGACTCCG |
|  |  | pCAMBIA1300-35S-*OsAIRP2*-YFPc-RP | TCTGCTTGTCCATCGGTGCACTAGTGTCGACCTTGATACGGTACTCGTAAATGTCA |
|  |  | pCAMBIA1300-35S-*OsAIRP2*-nYFP-FP | CGAGAGTCGATCTGATCAAAGAGACAGGATCCATGCGGAGGAGGTTCCAGGACTCCG |
|  |  | pCAMBIA1300-35S-*OsAIRP2*-nYFP-RP | CGAGCTCCTACATCGGTGCACTAGTGTCGACTCACTTGATACGGTACTCGTAAATG |
|  |  | pPR3-N-*OsAIRP2-FP* | CATTGGAAGTTGAATCTTCC |
|  |  | pPR3-N-*OsAIRP2-FP* | TCAACGCAGAGTGGCCATTA |
|  |  | pPR3-N-*OsAIRP2-RP* | CTTCAGGTTGTCTAACTCCT |

Table S2. Proteins prediction that may interact with OsPT4 by yeast two-hybrid sieve library.

| No. | Protein prediction | Sequence ID |
| --- | --- | --- |
| 1 | Oryza sativa Japonica Group E3 ubiquitin-protein ligase AIRP2 (LOC4348743), transcript variant X2, mRNA | XM_015757338.2 |
| 2 | Oryza sativa Japonica Group probable sugar phosphate/phosphate translocator At4g32390 (LOC4339191), mRNA | XM_015783824.2 |
| 3 | PREDICTED: Oryza sativa Japonica Group tricin synthase 2-like (LOC4345935), transcript variant X2, mRNA | XM_015794277.2 |
| 4 | >PREDICTED: Oryza sativa Japonica Group protein DETOXIFICATION 19, putative MATE efflux protein family protein (LOC4343318), mRNA | XM_015791806.2 |
| 6 | >PREDICTED: Oryza sativa Japonica Group GDSL esterase/lipase At5g45910 (LOC4340183), mRNA | XM_015787739.2 |
| 8 | >PREDICTED: Oryza sativa Japonica Group ubiquitin-conjugating enzyme E2 32 (LOC4332614), mRNA | XM_015775489.2 |
| 9 | >Oryza sativa Japonica Group probable aquaporin PIP2-7 (LOC4347729), mRNA | NM_001403935.1 |
| 10 | >PREDICTED: Oryza sativa Japonica Group glucan endo-1,3-beta-glucosidase GII (LOC4325938), mRNA | XM_015766957.2 |
